# Robust assembly of the aldehyde dehydrogenase Ald4p in *Saccharomyces cerevisiae*

**DOI:** 10.1242/bio.060070

**Published:** 2023-10-19

**Authors:** Channarong Nasalingkhan, Naraporn Sirinonthanawech, Chalongrat Noree

**Affiliations:** Institute of Molecular Biosciences, Mahidol University, 25/25 Phuttamonthon 4 Road, Salaya, Phuttamonthon, Nakhon Pathom 73170 Thailand

**Keywords:** Aldehyde dehydrogenase, Supramolecular assembly, Yeast

## Abstract

As part of our studies of yeast aldehyde dehydrogenase (Ald4p) assembly, we identified a population of transformants (SWORD strain) that show more robust filament formation of GFP-tagged Ald4p (Ald4p-GFP) than that of a wild type *ALD4::GFP* strain. Sequencing of the *ALD4* gene in the SWORD strain showed that the increased assembly was not due to changes to the *ALD4* coding sequence, suggesting that a second mutation site was altering Ald4p assembly. Using short-read whole-genome sequencing, we identified spontaneous mutations in *FLO9*. Introduction of the SWORD allele of *FLO9* into a wild-type *ALD4::GFP* yeast strain revealed that the changes to *FLO9* were a contributor to the increased length of Ald4p-GFP filaments we observe in the SWORD strain and that this effect was not due to an increase in Ald4p protein levels. However, the expression of the *FLO9* (SWORD) allele in wild-type yeast did not fully recapitulate the length control defect we observed in SWORD strains, arguing that there are additional genes contributing to the filament length phenotype. For our future work, this *FLO9* from SWORD will be tested whether it could show global effect, promoting the assembly of some other filament-forming enzymes.

## Teaser

**Summary:** A *FLO9* allele promotes self-assembly of GFP-tagged aldehyde dehydrogenase (Ald4p), suggesting a novel role in regulating the polymerization of Ald4p.

## INTRODUCTION

Aldehyde dehydrogenase increases cellular resistances to metabolic/chemical stress by catalyzing the conversion of aldehydes (e.g. acetaldehyde) into non-toxic products (e.g. acetate). *Saccharomyces cerevisiae* and most other organisms express both cytosolic and mitochondrial isoforms of aldehyde hydrogenases, allowing the cell to tune its response to stress (Wang et al., 1998; Aranda and del Olmo Ml, 2003; Navarro-Avino et al., 1999). Recently, a mitochondrial aldehyde dehydrogenase, Ald4p, was found to form high-order structures in yeast cells (Misonou et al., 2014; Noree, 2018; Noree et al., 2019a). Removal of its mitochondrial targeting sequence (MTS) allows Ald4p to polymerize into very long filaments in the cytoplasm of yeast cells. Furthermore, the ability of this retargeted Ald4p to form filaments is regulated by the availability of nutrients in the culture medium (Noree, 2018; Noree and Sirinonthanawech, 2020). These studies suggest that Ald4p filament assembly and enzyme activity are tightly coordinated (Noree and Sirinonthanawech, 2020).

In our previous structure-function studies of the relationship of Ald4p enzyme activity to Ald4p filament assembly (Noree and Sirinonthanawech, 2020), we observed a set of spontaneous clones (named ‘SWORD’ clones/strains) that have very long Ald4p-GFP filaments. As these clones resulted from our studies of Ald4p mutations, we assumed that we had unexpectedly generated novel *ALD4* mutations that increased filament assembly. However, sequencing of the coding sequence of chromosomal *ALD4* in these SWORD clones showed that the amino acid sequence of Ald4p was unaltered. This suggested that the SWORD clones had spontaneously acquired second mutation sites in genes that regulate Ald4p assembly. In order to identify such genes, we analyzed the genomic DNA samples of three SWORD clones and the original *ALD4::GFP* strain by short-read whole-genome sequencing (WGS). Analysis of the WGS data showed that the flocculation gene *FLO9* (Goossens et al., 2015) had very high structure variations. In this report, we tested whether *FLO9* derived from a SWORD clone could stimulate the assembly of Ald4p-GFP in yeast cells. Our work has revealed an interesting finding about *FLO9* that might be applied to the manipulation of Ald4p assembly and activity control.

## RESULTS AND DISCUSSION

### The increased length of Ald4p-GFP filaments in SWORD clones is not due to increased protein expression of Ald4p

**Fig. 1.**
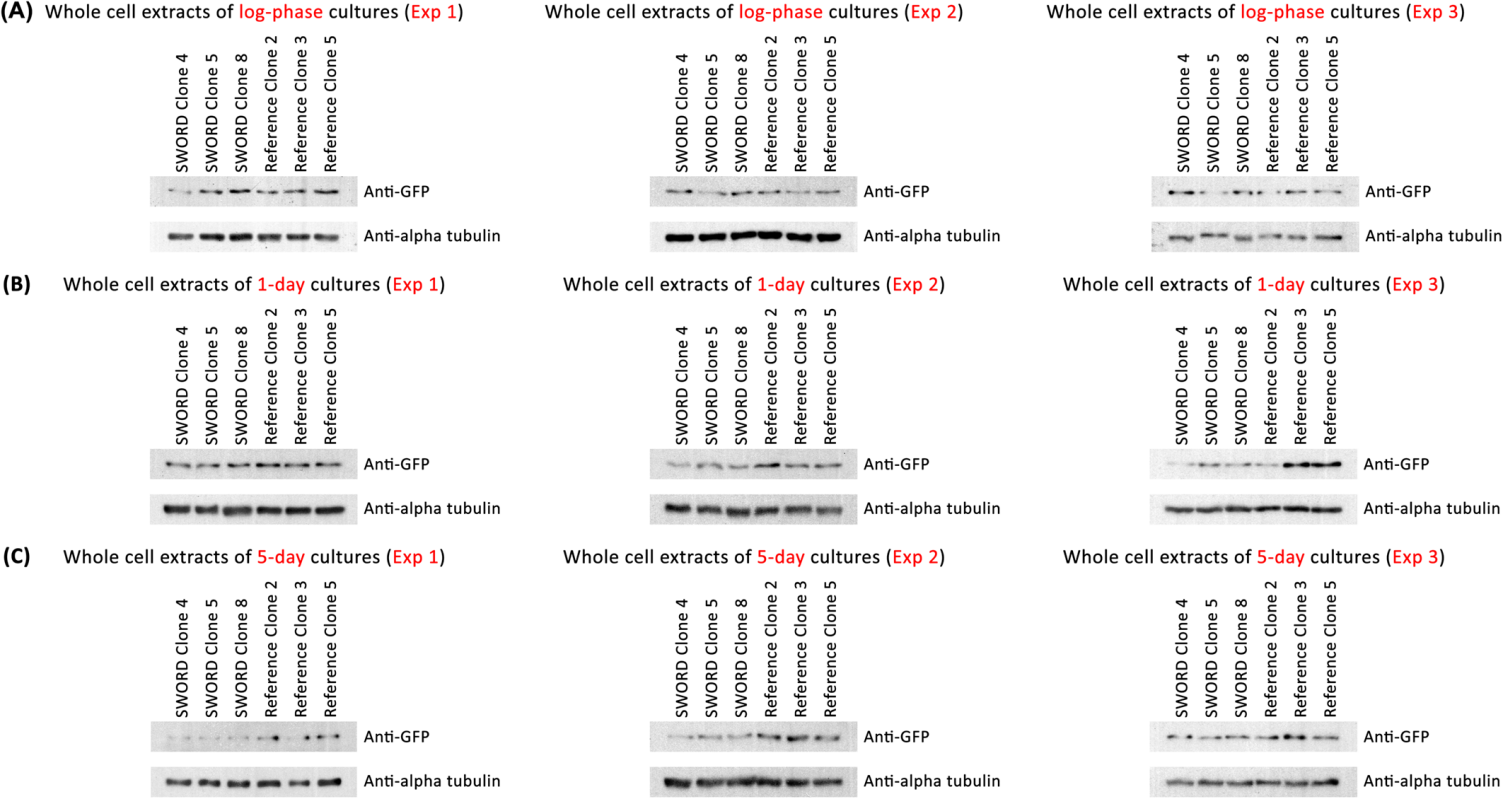
**Robust assembly of Ald4p-GFP, found in SWORD clones, is not caused by its protein levels.** Western blot analysis of Ald4p-GFP levels (SWORD versus reference *ALD4::GFP* constructs) was performed by using whole protein extracts prepared from the cells grown to log-phase (A), saturation (1-day cultures) (B) and stationary phase (5-day cultures) (C). Full blots are presented in Figs S1-S3. The anti-GFP antibody was used to detect the GFP-tagged Ald4p, whereas the anti-α-tubulin antibody was used to detect the internal loading control (α-tubulin). Three independent experiments were performed for each time point. The contrast was adjusted here only for visualization purpose. The original blots (without image contrast adjustment) were quantified by ImageJ. After normalization with the loading control (α-tubulin), Ald4p-GFP levels of the SWORD clones were expressed as fold-change values, relative to those of the reference *ALD4::GFP* clones (Table S2).

One common way to increase filament length is to increase expression of the filament-forming protein. In order to test whether the increased length of Ald4p-GFP filaments in SWORD clones is due to increased expression of Ald4p-GFP, we analyzed Ald4p-GFP protein levels in SWORD and wild-type strains by western blot analysis using the protein samples extracted from yeast cells cultured to log phase, saturation (1-day cultures) and stationary phase (5-day cultures). After normalization with the loading control (α-tubulin), we found that the Ald4p-GFP levels in SWORD clones were less than those of the reference clones (0.94-, 0.57- and 0.47-fold for the cells grown to log-phase, saturation and stationary phase, respectively) (Fig. 1; Figs S1-S3, Table S2). This result argues that the increase in filament length is not a secondary effect of increasing Ald4p-GFP expression and that it might be due to altered regulation of filament assembly. Thus, the changes in the SWORD strains are more similar to previously studied mutations in other filament-forming enzymes that increase filament formation without increasing protein levels (Noree et al., 2014; Noree, 2018). This result is also consistent with altered sensitivity to the physiological, physicochemical and metabolic states within the cells as well as environmental stimuli that regulate filament formation (Narayanaswamy et al., 2009; Noree et al., 2019a; Hansen et al., 2021; Petrovska et al., 2014; Noree et al., 2010; Aughey and Liu, 2015; Barry et al., 2014).

### WGS suggests that *FLO9* might be responsible for robust assembly of the yeast aldehyde dehydrogenase Ald4p

**Fig. 2.**
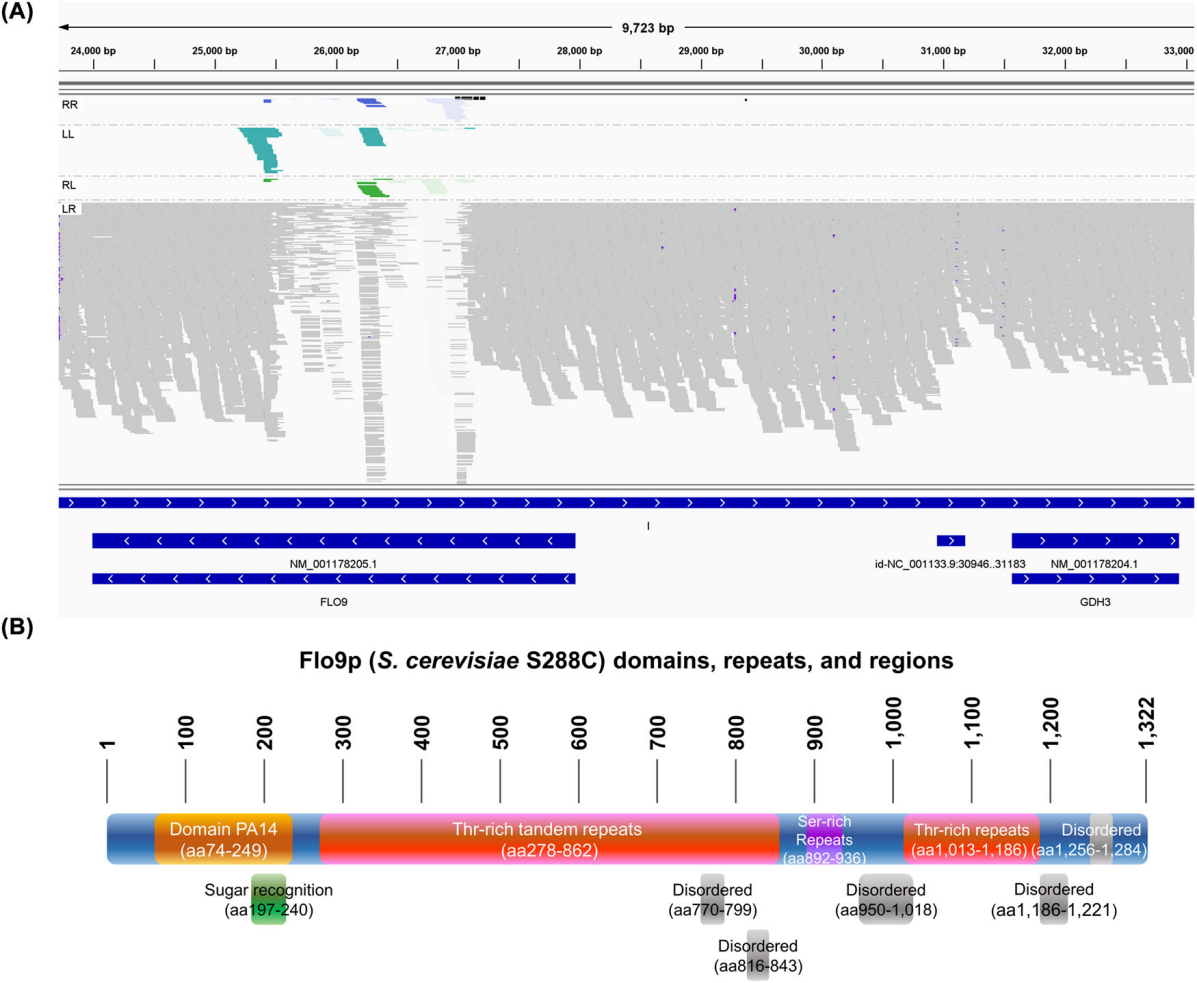
**Short-read WGS bioinformatic analysis suggests that *FLO9* might be responsible for robust assembly of Ald4p-GFP.** (A) Structure variations found in *FLO9*. The Integrative Genomics Viewer by Broad Institute, was used to display read mapping. LR are normal reads (grey bars), RL implies duplication or translocation (green bars), LL and RR imply inversion (turquoise and blue bars, respectively). (B) Flo9p (*S. cerevisiae* S288C, UniProtKB accession number P39712) contains a lot of repeats and disordered regions. Nucleotide and amino acid sequence alignments of *S. cerevisiae* S288C and BY4741 are shown in Figs S4 and S5, respectively.

As the SWORD clones had no changes in the *ALD4* gene, the SWORD phenotype was most likely due to spontaneous changes in genes that regulate Ald4p assembly. To identify these candidate regulatory genes, we performed short-read WGS on genomic DNA samples from SWORD and reference *ALD4::GFP* yeast strains. WGS bioinformatic analysis identified *FLO9* as one of the genes showing high structure variations (Fig. 2A). Moreover, analysis of three different SWORD clones found that *FLO9* was the only gene initially identified to have single-nucleotide polymorphism (SNP) that was shared by all three clones. This SNP causes two amino acid changes, Asn374Thr and Ser375Gly, in the *FLO9* coding sequence. While this finding led us to explore the role of *FLO9* in regulating filament length, our subsequent resequencing of *FLO9* in wild-type and SWORD clones revealed that this SNP was shared by both SWORD clones and wild-type stains and that it was a false-positive SNP.

**Fig. 3.**
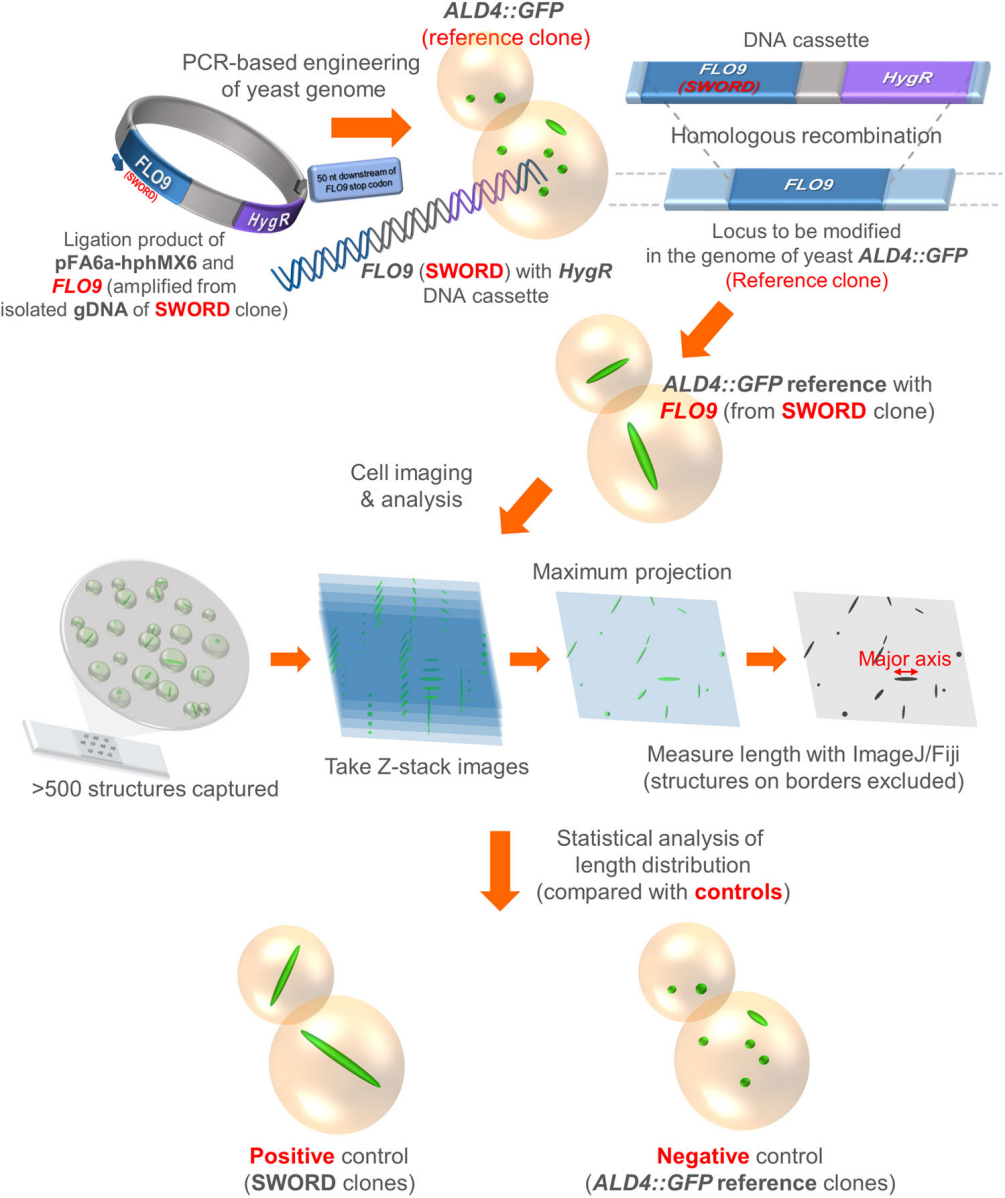
**Experimental design for introducing *FLO9* amplified from a SWORD clone into the *FLO9* chromosomal locus in the genome of yeast *ALD4::GFP* reference and comparative analysis of length distribution of Ald4p-GFP.** Ligation reaction of pFA6a-hphMX6 and *FLO9* insert (amplified from the genomic DNA sample of a SWORD clone) was directly used as the PCR template for preparing the DNA cassette (*FLO9* from SWORD and the hygromycin resistance gene). The purified DNA cassette was introduced into the genome of yeast *ALD4::GFP* reference to replace the original *FLO9* gene with the *FLO9* allele from SWORD. After verification by PCR and Sanger DNA sequencing, the images of resulting yeast construct were analyzed for length distribution of Ald4p-GFP, compared to those of SWORD (positive control) and *ALD4::GFP* reference (negative control).

Typically, *FLO9* (3969 nucleotides) codes for the cell wall protein ‘flocculin’ (Flo9p, 1322 amino acids, about 138 kDa), which is responsible for reversible cell-to-cell adhesion and aggregation, so-called ‘flocculation’ (Verstrepen et al., 2003; Goossens et al., 2015). In this report, we wanted to test whether *FLO9* could have an impact on the assembly of yeast Ald4p (Fig. 3).

### Replacing the original *FLO9* with its counterpart derived from a SWORD clone can make the Ald4p-GFP structures longer

Motivated by our SNP analysis, we made a new yeast construct by introducing the DNA cassette of *FLO9*, amplified from the genomic DNA sample of a SWORD clone, along with a hygromycin resistance gene into the genome of a reference *ALD4::GFP* clone. Initially, we focused on subcloning *FLO9* from SWORD strains into the plasmid pFA6a-hphMX6. However, we could not generate a recombinant plasmid with full-length *FLO9* as the sequence in the middle of the gene, where tandem repeats are located, always disappeared during the bacterial transformation process. A previous report has demonstrated that these repeats, often found in the genes coding for cell wall proteins, can trigger recombination events within the gene itself or with any pseudogene (Verstrepen et al., 2005); thus, the number of repeats and disordered regions within *FLO* genes could be varied between different strains of *S. cerevisiae*. Consistent with our finding, the nucleotide and amino acid sequences of *S. cerevisiae* S288C and BY4741 (a direct descendent of S288C and used as the background strain to create all yeast constructs in this study and our previous studies) are different (92.9% identical for their nucleotide sequences and 93% identical for their amino acid sequences), especially in the repeats and disordered regions (Fig. 2B; Figs S4 and S5). After several attempts and failures, we decided to use the ligation reaction between pFA6a-hphMX6 and the *FLO9* insert (derived from SWORD) directly as the template DNA for PCR, and we could successfully prepare the DNA cassette of *FLO9* (from SWORD) and the hygromycin resistance gene. This DNA cassette was transformed into the yeast *ALD4::GFP* reference clone in order to investigate whether the *FLO9* (from SWORD) could have an impact on Ald4p-GFP assembly.

After strain verification by PCR and DNA sequencing, a few different clones of the new yeast construct were subjected to live-cell imaging, along with SWORD (as the positive control) and the reference *ALD4::GFP* (as the negative control) (Fig. 3). In this study, the way we prepared the yeast samples for imaging was different from our previous studies with other filament-forming enzymes. Similar to a recent study (Krzek et al., 2022), we noticed that the filament length of Ald4p-GFP is sensitive to shaking conditions (unpublished data). In order to circumvent this problem, we scraped the cells directly from the agar plate, resuspended them in 1× PBS, prepared a wet slide, and imaged the filaments by fluorescence microscopy (the whole process was limited to 30 min for each prep).

**Fig. 4.**
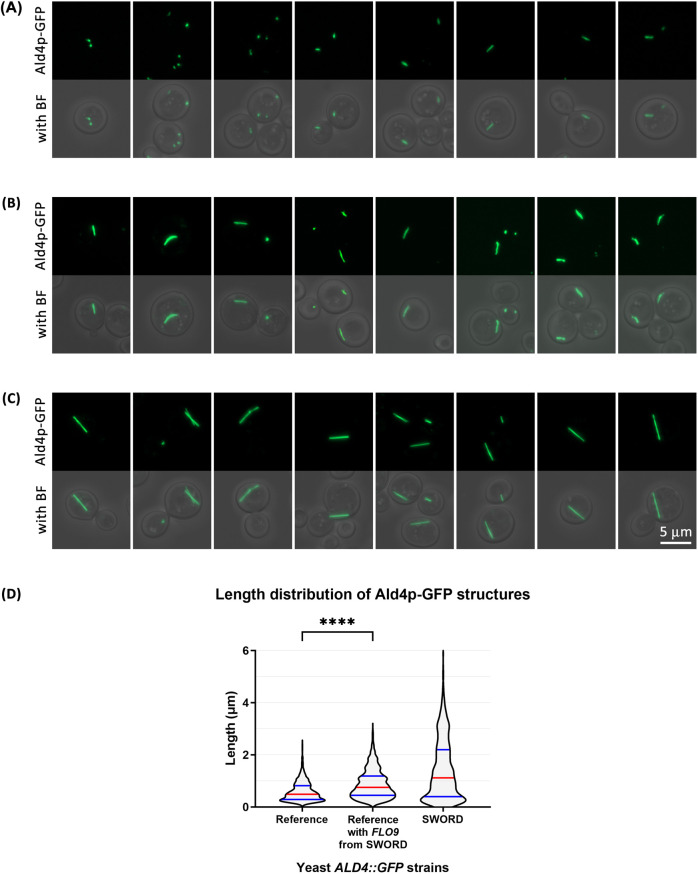
**Yeast *ALD4::GFP* reference cells getting *FLO9* allele from SWORD showed a significant increase in the length of their Ald4p-GFP structures.** (A-C) Fluorescence live-cell images (BF, bright-field) of yeast *ALD4::GFP* reference (negative control) (A), yeast *ALD4::GFP* reference with *FLO9* derived from SWORD (B) and yeast *ALD4::GFP* ‘SWORD’ (positive control) (C). (D) Length distribution of the Ald4p-GFP structures shown in a violin plot (the red line represents median, blue lines represent quartiles, and **** indicates statistically significant difference between two groups with *P*<0.0001, using an unpaired, non-parametric, Kolmogorov–Smirnov test). The length distribution summary (showing the number of structures analyzed) and the raw data (collected from at least three independent experiments) are presented in Table 1 and Table S3, respectively.

Our analysis found that the average length of Ald4p-GFP structures in the new yeast construct (reference *ALD4::GFP* with *FLO9* derived from SWORD) was 0.8771±0.5431 μm (average±s.d.), whereas Ald4p-GFP structures found in SWORD (positive control) and the reference *ALD4::GFP* (negative control) were, on average, 1.421±1.151 and 0.5904±0.3719 μm long, respectively (Fig. 4, Tables 1 and 2). According to the significant difference (*P*<0.0001) in the length distribution of Ald4p-GFP structures between the new yeast construct and the reference *ALD4::GFP*, alterations at the *FLO9* locus alter the assembly of Ald4p-GFP. However, as the length distribution of Ald4p-GFP structures of the new construct is not comparable to that of SWORD clones, *FLO9* is not the sole genetic factor regulating Ald4p-GFP filaments in the yeast cells. This is consistent with our resequencing results that indicated that the *FLO9* Asn374Thr and Ser375Gly double-mutation SNP was not unique to SWORD clones.

**Table 1.**
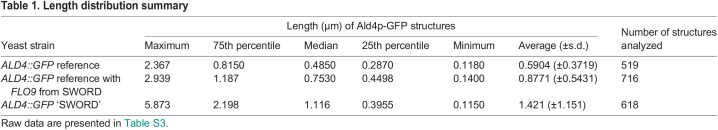
Length distribution summary

**Table 2.**
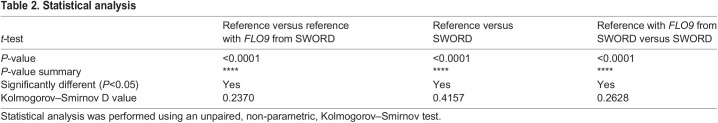
Statistical analysis

Although we were led to *FLO9* via a false positive, our studies indicate that alterations at the *FLO9* locus do significantly alter Ald4p filament length. The selectable marker – hygromycin resistance gene or hygR – was tested not be an effector for Ald4p filament assembly as the *flo9*Δ*::hygR* yeast construct showed a phenotype similar to that of the wild-type *ALD4::GFP* strain (unpublished data). The mechanism underlying this is unclear but could be due to additional uncharacterized SNPs outside of the coding sequence and/or changes of chromosome structure around *FLO9* locus, such as chromosomal inversion, translocation, or even a large DNA insertion and/or deletion that can be better identified by long-read sequencing (Hiatt et al., 2021).

Future studies directed at determining whether *FLO9* from SWORD has a stimulatory effect on other known filament-forming cytosolic enzymes, such as CTP synthetase (Ura7/8p) (Noree et al., 2010) and asparagine synthetase (Asn1/2p) (Narayanaswamy et al., 2009; Shen et al., 2016; Noree et al., 2019b), or mitochondrial enzymes, such as acetyl-CoA carboxylase (Acc1p) and threonine dehydratase (Ilv1/2p) (Noree et al., 2019a), in *S. cerevisiae* will be quite revealing. Furthermore, functional and localization studies of Flo9p in SWORD and reference *ALD4::GFP* strains will help determine how Flo9p is altered in SWORD strains and how that contributes to regulating enzyme structures.

According to a study by Frieman and Cormack (2004), it has been suggested that serine and threonine residues within the repeats of Flo9p are important for the protein to be targeted to the outer cell wall. If some of these residues are altered and make the protein unable to be directed to the cell surface, but rather the protein accumulates inside the cells, the *FLO9* variants might display a novel function, probably being involved in supramolecular assembly of certain proteins or enzymes. If so, *FLO9* and its variable gene products could be very intriguing for further studies and applications.

## MATERIALS AND METHODS

### Bacterial and yeast strains

*Escherichia coli* One Shot™ MAX Efficiency™ DH5α-T1R competent cells (Thermo Fisher Scientific, USA) were used for cloning and propagation of pFA6a-FLO9(SWORD)-hphMX6 (cloned from pFA6-hphMX6, EUROSCARF, Scientific Research and Development GmbH, Germany). Bacterial cultures were maintained in LB medium [1% (w/v) Bacto™ tryptone (BD Biosciences), 0.5% (w/v) Bacto™ yeast extract (BD Biosciences) and 1% (w/v) NaCl (Merck)], supplemented with ampicillin (100 µg/ml) (PanReac AppliChem) at 37°C.

Yeast *ALD4::GFP; kanR* (*S. cerevisiae* BY4741 used as background strain) was constructed in our previous study (Noree and Sirinonthanawech, 2020) and used as the base strain in this study to create a new yeast strain *ALD4::GFP; kanR* with *FLO9(SWORD); hygR*. Yeast cultures were maintained in YPD medium [(2% (w/v) Bacto™ peptone (BD Biosciences), 1% (w/v) Bacto™ yeast extract and 2% (w/v) glucose (Sigma-Aldrich)] at 30°C. G418 (400 µg/ml) (PanReac AppliChem) and hygromycin B (200 µg/ml) (Merck) were used for selection of the corresponding yeast strains.

### Short-read WGS

The genomic DNA samples of yeast *ALD4::GFP* ‘SWORD’ (three clones) and yeast *ALD4::GFP* reference (one clone) were prepared using TIANamp Yeast DNA Kit (TIANGEN). They were then sent out for short-read WGS (NovaSeq 6000, Novogene, Singapore). The bioinformatic analysis of WGS data was performed by Ward Medic (Thailand). The WGS data were deposited in the National Center for Biotechnology Information (NCBI) Sequence Read Archive (SRA) with the SRA numbers SRR23883646 (for SWORD4), SRR23883645 (for SWORD5), SRR23883644 (for SWORD8) and SRR23883643 (for *ALD4::GFP* reference).

### Western blot analysis

Whole-cell lysates of two yeast strains, SWORD and reference *ALD4::GFP* (three different clones for each), were prepared by growing them in YPD broth to three growth stages; log-phase, saturation (or 1-day culture) and stationary phase (or 5-day culture). For log-phase cultures, one OD_600_ cells were collected, whereas five OD_600_ cells and ten OD_600_ cells were collected for saturation and stationary-phase cultures, respectively. Then, the collected cells were resuspended in 100 µl 1× SDS-PAGE sample buffer containing 4 M urea, 1:20 β-mercaptoethanol (PanReac AppliChem) and 1:1000 protease inhibitor cocktail (Sigma-Aldrich). About 50 µl glass beads (425-600 µm) (Sigma-Aldrich) were added to each sample before vortexing it vigorously for 1-2 min. The protein samples were boiled at 95°C for 5 min, immediately placed on ice for 5 min, centrifuged at 9391 ***g*** for 1 min, and kept at −25°C until use. SDS-PAGE (8% separating gel) was performed, and the BLUeye Prestained Protein Ladder (Sigma-Aldrich) was used to estimate the size of the resolved proteins. After running SDS-PAGE, the separated proteins were then transferred from polyacrylamide gels to PVDF membranes using the Trans-Blot^®^ Turbo™ Transfer System (Bio-Rad). Each blot was split into two pieces between the 75 and 63 kDa bands of the prestained ladder. Western blotting was performed following standard protocols. For protein detection, the upper half of the membrane was incubated with 1:5000 rabbit anti-GFP polyclonal antibody (A01388, GenScript) (to detect GFP-tagged Ald4p), followed by incubation with 1:5000 HRP-conjugated goat anti-rabbit IgG (31460, Thermo Fisher Scientific). The lower half of the membrane was incubated with 1:5000 mouse anti-α-tubulin monoclonal antibody (12G10, Developmental Studies Hybridoma Bank) (to detect α-tubulin as the loading control), followed by incubation with 1:5000 HRP-conjugated goat anti-mouse IgG (62-6520, Thermo Fisher Scientific). Both the upper and lower halves of each blot were subsequently re-assembled before developing the chemiluminescent signals with Amersham™ ECL™ Western Blotting Analysis System (GE Healthcare). ImageJ/Fiji (Schneider et al., 2012) was used to quantitate the intensity of protein bands in order to compare the normalized expression of GFP-tagged Ald4p in the SWORD versus reference *ALD4::GFP* yeast clones.

### Construction of pFA6a-FLO9(SWORD)-hphMX6

The coding sequence of the *FLO9* gene was amplified by PCR using the KOD Hot Start DNA Polymerase Kit (Merck). The genomic DNA isolated from a SWORD clone was used as the DNA template. The PCR product was purified using GenepHlow^TM^ Gel/PCR Kit (Geneaid) and then cloned into pFA6a-hphMX6 (EUROSCARF, Scientific Research and Development GmbH, Germany) at the *Hin*dIII and *Sma*I recognition sites. *Hin*dIII and *Sma*I restriction digests were performed according to the manufacturer's instructions (Thermo Fisher Scientific). The ligation was performed using T4 DNA Ligase Kit (New England Bioloabs). After selection on LB agar supplemented with ampicillin, the recombinant plasmid was extracted using the Presto™ Mini Plasmid Kit (Geneaid) from each randomly selected bacterial transformant for verification by Sanger DNA sequencing (Macrogen). The primers used for cloning and DNA sequencing are shown in Table S1.

### Construction of yeast *ALD4::GFP; kanR* with *FLO9(SWORD); hygR*

After several attempts, we could not obtain the recombinant plasmid of pFA6a-hphMX6 with full-length *FLO9* (amplified from SWORD's genomic DNA); therefore, we decided to use the ligation products directly as the PCR template instead. The DNA cassette, harboring *FLO9* derived from SWORD and the hygromycin resistance gene, was successfully prepared by PCR using CN0087 and CN0060 as forward and reverse primers, respectively (Table S1). After PCR purification, the DNA cassette was transformed into yeast *ALD4::GFP* reference (Noree and Sirinonthanawech, 2020) using lithium acetate and the heat-shock method (Petracek and Longtine, 2002) with some modifications. Briefly, competent yeast cells were freshly prepared by growing the cells (30-ml culture) to log phase at 30°C with shaking. The cells were harvested at 3000 ***g*** for 5 min and washed once with sterile water, before being resuspended in 400 µl of a solution containing 100 mM lithium acetate (Sigma-Aldrich) and 1× TE (10 mM Tris pH 8.0 and 1 mM EDTA) to allow them to become competent cells. After incubation at room temperature for 10 min, 100 µl of yeast competent cell suspension was added to the tube with the whole purified DNA cassette and 100 µg single-stranded DNA (Sigma-Aldrich), followed by the addition of 600 µl of a solution containing 100 mM lithium acetate, 1× TE and 40% (w/v) polyethylene glycol 3350 (Sigma-Aldrich). The transformation reaction was incubated at 30°C with shaking for 45 min, then the heat shock was performed at 42°C for 30 min. After placing on ice for 5 min, the cells were collected by centrifugation at 3381 ***g*** for 2 min, and then resuspended in 1× PBS before spreading onto the YPD agar plates. After selection on YPD agar supplemented with hygromycin B, the genomic DNA was then extracted using STES buffer [0.2 M Tris pH 7.6, 0.5 M NaCl, 0.1% (w/v) SDS and 0.01 M EDTA)] from each randomly selected yeast transformant to be used as the PCR template in order to get the PCR product for further verification by Sanger DNA sequencing. All the primers used for preparing the DNA cassette, preparing the PCR product for DNA sequencing, and the sequencing primers are shown in Table S1.

### Yeast cell imaging and Ald4p-GFP length distribution analysis

Yeast samples of (1) *ALD4::GFP* reference, transformed with the DNA cassette containing *FLO9* (from SWORD) and the hygromycin resistance gene, (2) *ALD4::GFP* reference (as the negative control) and (3) SWORD (as the positive control) were prepared by first scraping the cells from their agar plate and resuspending them in a microcentrifuge tube containing 1 ml of 1× PBS (Merck). The cell suspension (about 8-10 µl) was dropped onto a microscope slide (Shandon SuperFrost Plus, Thermo Fisher Scientific), followed by placing a coverslip over the sample (Menzel Gläser, Thermo Fisher Scientific). The slide was then put upside down onto a lint-free lab wipe and gently pressed to remove the excess liquid and to help prevent the cells from floating around. The imaging was performed using Zeiss Axio Imager.Z2 and ApoTome.2 with EC Plan_NEOFLUAR 100×/1.3 oil objective lens. *Z*-stack images were subjected to ‘ApoTome’ processing and compressed into a single image with maximum projection using ZEN 3.1 (blue edition). Length measurement of Ald4p-GFP structures was performed using ImageJ/Fiji. After opening an image, the ‘Set Scale’ function under the menu bar ‘Analyze’ was set to be 22.0264 pixels/μm (specific for images taken by Zeiss Axio Imager.Z2 and ApoTome.2 with 100× objective lens). Under ‘Analyze’ and ‘Set Measurements’, ‘Area’, ‘Fit ellipse’ and ‘Display label’ were selected. Next, under ‘Image’ and ‘Type’, ‘8-bit’ was selected. Then, under ‘Image’ and ‘Adjust’, the threshold with a setting defined by ‘Otsu’ (in the drop-down list) was chosen. After that, under ‘Process’ and ‘Binary’, ‘Convert to Mask’ was selected. Subsequently, under ‘Analyze’, ‘Analyze Particles’ was selected, the number ‘0.01-infinity’ was put in the ‘Size (μm^2)’ box (to exclude structures with size <0.01 μm^2^ from the analysis), ‘Outlines’ was chosen in the ‘Show’ box, and the ‘Display results’, ‘Summarize’ and ‘Exclude on edges’ checkboxes were ticked. After the ‘Results’ window showed up, the value of the ‘major axis’ of each Ald4p-GFP structure was collected for length distribution analysis. Statistical analysis (unpaired *t*-test; non-parametric, Kolmogorov–Smirnov test) was performed using GraphPad Prism 9 Version 9.5.1 (733).

## Supplementary Material

10.1242/biolopen.060070_sup1 Supplementary information
