# Supplementary material for: Robust assembly of the aldehyde dehydrogenase Ald4p in Saccharomyces cerevisiae
Source: Biol Open. 2023 Oct 19;12(10):bio060070. doi: 10.1242/bio.060070 (PMC10602002; doi:10.1242/bio.060070)
Supplement: Supplementary information [file biolopen-12-060070-s1.pdf]

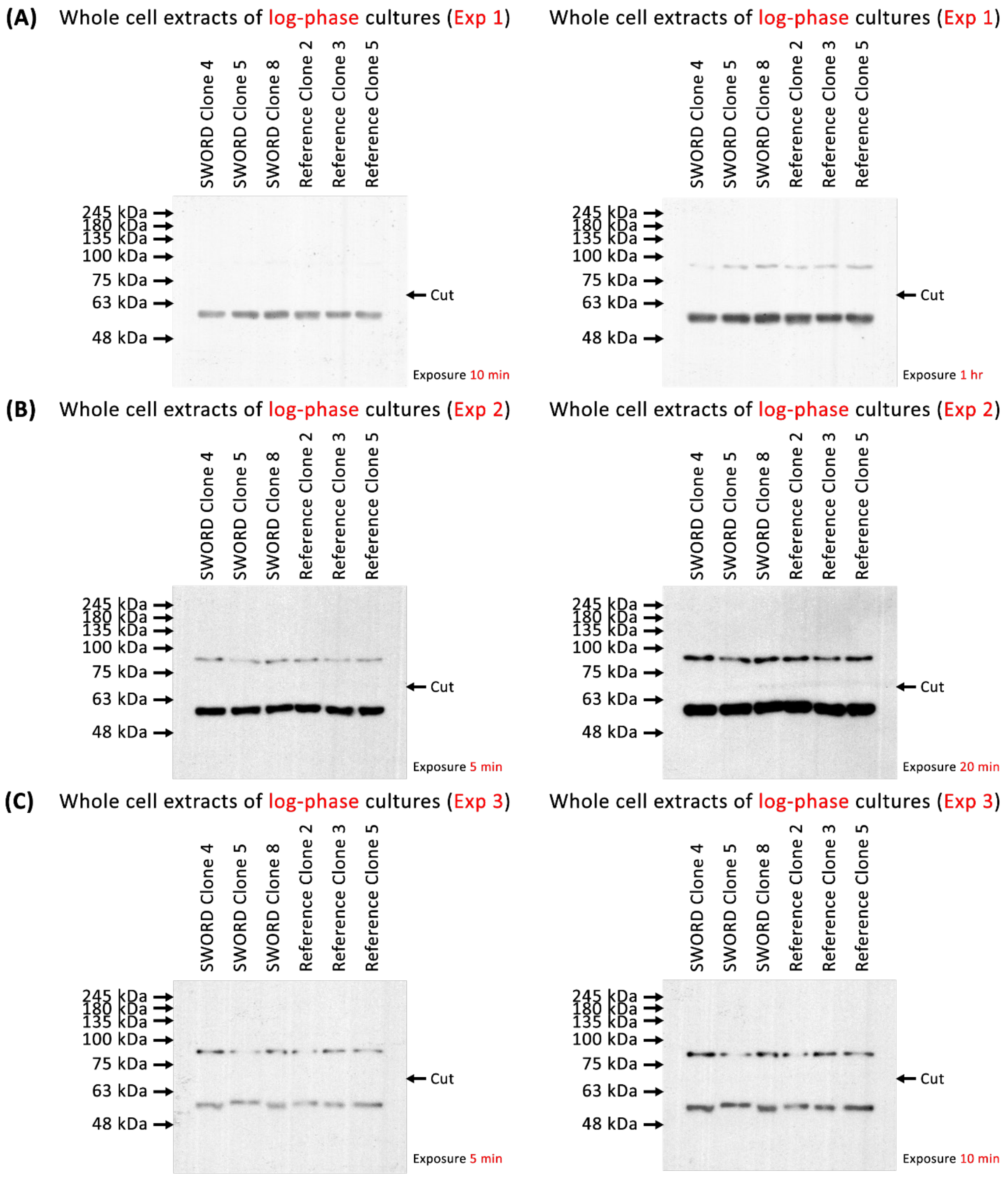

Fig. S1. Full blots of log-phase yeast cultures (SWORD vs. reference *ALD4::GFP* clones). Three independent experiments (**A**) – (**C**) were performed; left and right panels showing blots with different exposure time points.

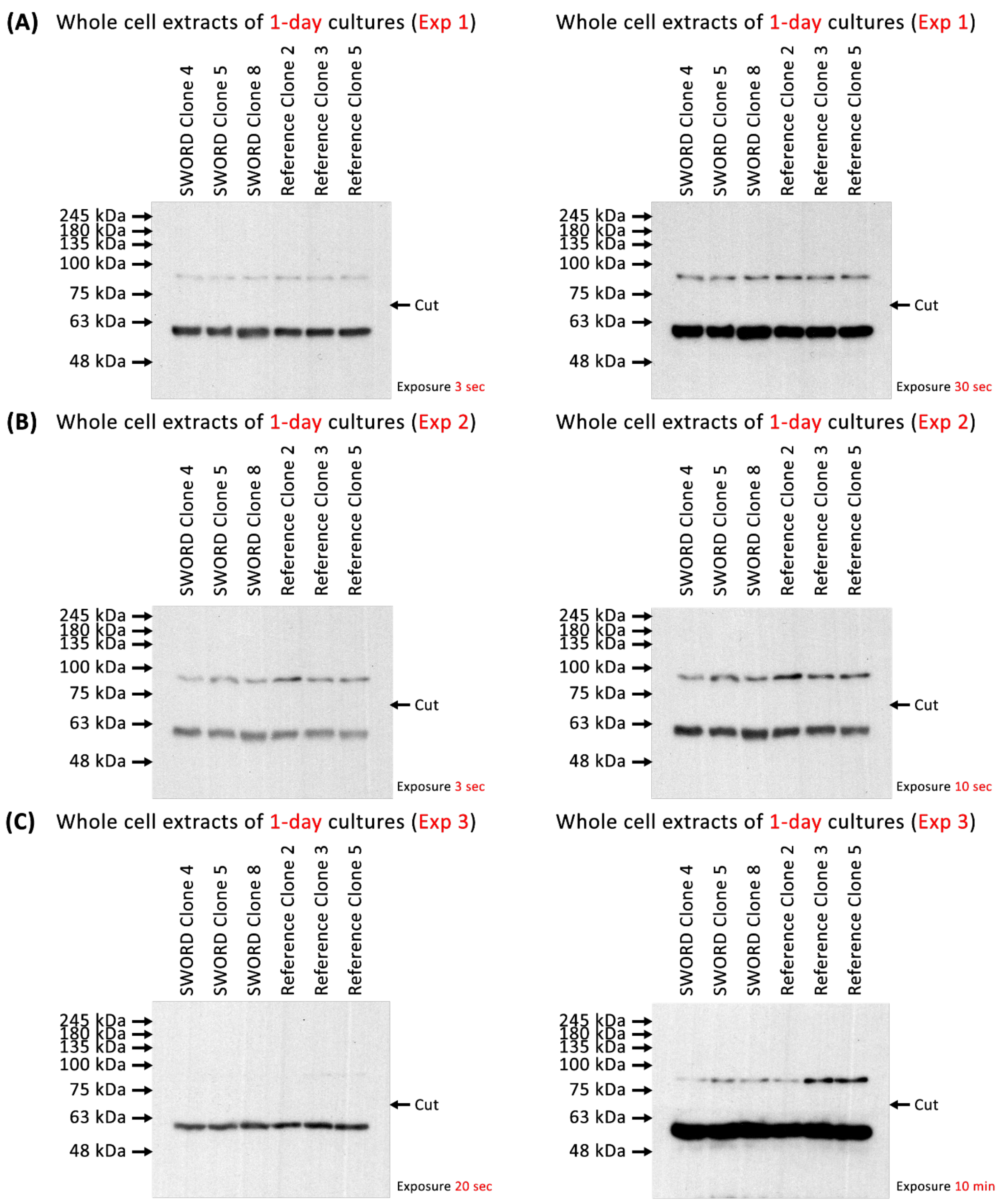

Fig. S2. Full blots of 1-day yeast cultures (SWORD vs. reference *ALD4::GFP* clones). Three independent experiments (**A**) – (**C**) were performed; left and right panels showing blots with different exposure time points.

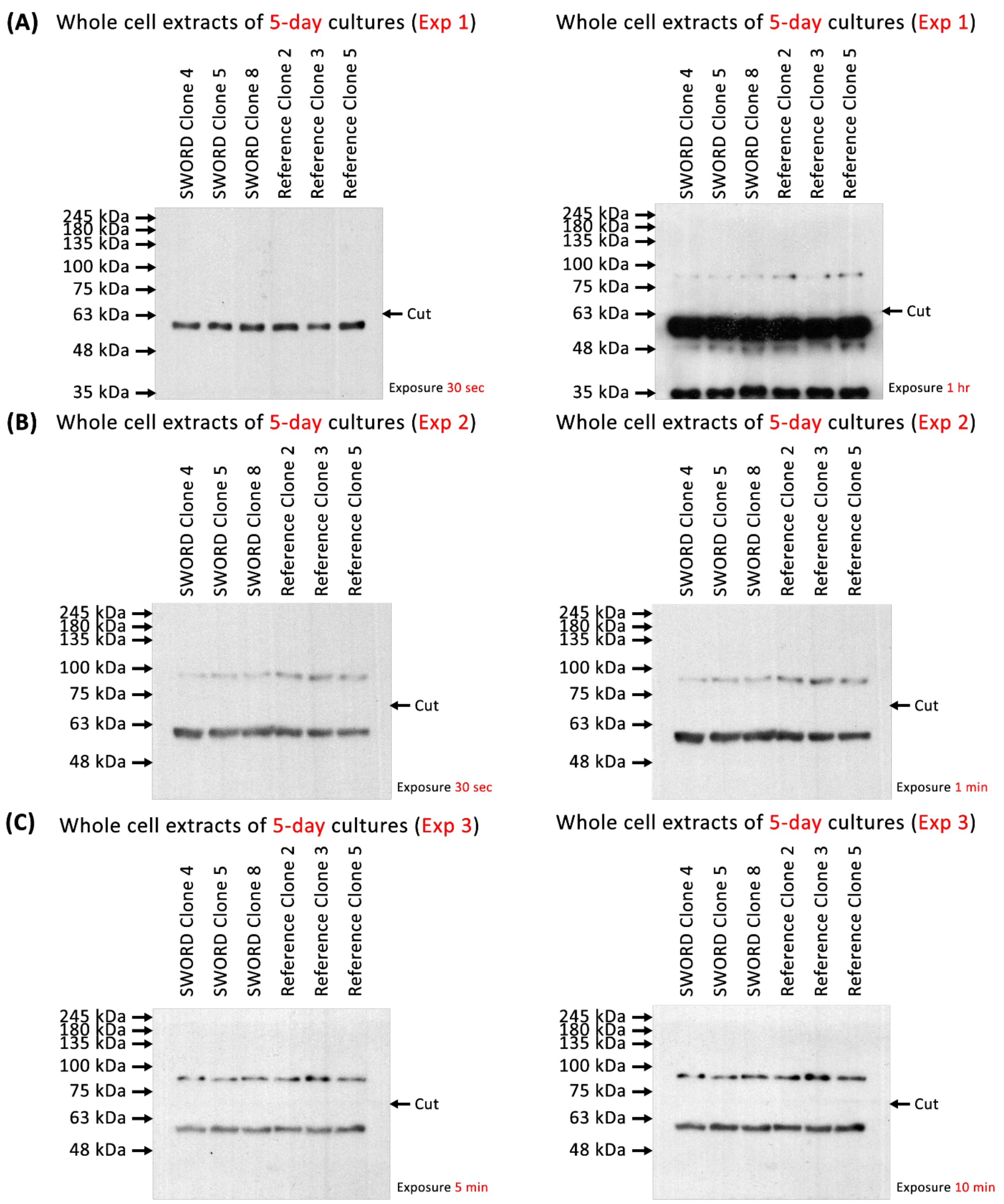

Fig. S3. Full blots of 5-day yeast cultures (SWORD vs. reference *ALD4::GFP* clones). Three independent experiments (**A**) – (**C**) were performed; left and right panels showing blots with different exposure time points.

Biology Open • Supplementary information

```
FLO9_S288C          1 ATGTCTCTGGCACATTATTGTTTACTACTAGCCATCGTCACATTGCTGGG      50
                      ||||||||||||||||||||||||||||||||||||||||||||||||||
FLO9_BY4741         1 ATGTCTCTGGCACATTATTGTTTACTACTAGCCATCGTCACATTGCTGGG      50

FLO9_S288C         51 ATTAACTAATGTTGTCTCTGCGACTACAGCGGCATGCCTGCCAGCAAACT     100
                      ||||||||||||||||||||||||||||||||||||||||||||||||||
FLO9_BY4741        51 ATTAACTAATGTTGTCTCTGCGACTACAGCGGCATGCCTGCCAGCAAACT     100

FLO9_S288C        101 CAAGGAAGAATGGTATGAATGTAAACTTTTACCAGTATTCATTGAGAGAT     150
                      ||||||||||||||||||||||||||||||||||||||||||||||||||
FLO9_BY4741       101 CAAGGAAGAATGGTATGAATGTAAACTTTTACCAGTATTCATTGAGAGAT     150

FLO9_S288C        151 TCCTCCACATATTCGAATGCAGCATATATGGCTTATGGATATGCCTCAAA     200
                      ||||||||||||||||||||||||||||||||||||||||||||||||||
FLO9_BY4741       151 TCCTCCACATATTCGAATGCAGCATATATGGCTTATGGATATGCCTCAAA     200

FLO9_S288C        201 AACTAAACTGGGTTCTGTCGGAGGACAAACTGATATCTCGATTGATTATA     250
                      ||||||||||||||||||||||||||||||||||||||||||||||||||
FLO9_BY4741       201 AACTAAACTGGGTTCTGTCGGAGGACAAACTGATATCTCGATTGATTATA     250

FLO9_S288C        251 ATATTCCTTGTGTTAGTTCATCAGGCACATTTCCTTGTCCTCAAGAAGAT     300
                      ||||||||||||||||||||||||||||||||||||||||||||||||||
FLO9_BY4741       251 ATATTCCTTGTGTTAGTTCATCAGGCACATTTCCTTGTCCTCAAGAAGAT     300

FLO9_S288C        301 TTATATGGTAATTGGGGATGCAAAGGAATTGGTGCTTGTTCTAATAATCC     350
                      ||||||||||||||||||||||||||||||||||||||||||||||||||
FLO9_BY4741       301 TTATATGGTAATTGGGGATGCAAAGGAATTGGTGCTTGTTCTAATAATCC     350

FLO9_S288C        351 AATAATTGCATACTGGAGTACTGATTTATTTGGTTTCTATACTACCCCAA     400
                      ||||||||||||||||||||||||||||||||||||||||||||||||||
FLO9_BY4741       351 AATAATTGCATACTGGAGTACTGATTTATTTGGTTTCTATACTACCCCAA     400

FLO9_S288C        401 CAAACGTAACCCTAGAAATGACAGGTTATTTTTTACCACCACAGACGGGT     450
                      ||||||||||||||||||||||||||||||||||||||||||||||||||
FLO9_BY4741       401 CAAACGTAACCCTAGAAATGACAGGTTATTTTTTACCACCACAGACGGGT     450

FLO9_S288C        451 TCTTACACATTCAAGTTTGCTACAGTTGACGACTCTGCAATTCTATCAGT     500
                      ||||||||||||||||||||||||||||||||||||||||||||||||||
FLO9_BY4741       451 TCTTACACATTCAAGTTTGCTACAGTTGACGACTCTGCAATTCTATCAGT     500

FLO9_S288C        501 CGGTGGTAGCATTGCGTTCGAATGTTGTGCACAAGAACAACCTCCCATCA     550
                      ||||||||||||||||||||||||||||||||||||||||||||||||||
FLO9_BY4741       501 CGGTGGTAGCATTGCGTTCGAATGTTGTGCACAAGAACAACCTCCCATCA     550

FLO9_S288C        551 CGTCGACTAACTTCACCATCAATGGTATCAAGCCATGGAATGGAAGTCCC     600
                      ||||||||||||||||||||||||||||||||||||||||||||||||||
FLO9_BY4741       551 CGTCGACTAACTTCACCATCAATGGTATCAAGCCATGGAATGGAAGTCCC     600

FLO9_S288C        601 CCTGATAATATTACAGGGACTGTCTACATGTATGCTGGTTTCTATTATCC     650
                      ||||||||||||||||||||||||||||||||||||||||||||||||||
FLO9_BY4741       601 CCTGATAATATTACAGGGACTGTCTACATGTATGCTGGTTTCTATTATCC     650

FLO9_S288C        651 AATGAAGATTGTTTACTCAAATGCCGTTGCCTGGGGTACACTTCCAATTA     700
                      ||||||||||||||||||||||||||||||||||||||||||||||||||
FLO9_BY4741       651 AATGAAGATTGTTTACTCAAATGCCGTTGCCTGGGGTACACTTCCAATTA     700

FLO9_S288C        701 GTGTGACACTACCAGATGGCACTACCGTTAGTGATGACTTTGAAGGGTAC     750
                      ||||||||||||||||||||||||||||||||||||||||||||||||||
FLO9_BY4741       701 GTGTGACACTACCAGATGGCACTACCGTTAGTGATGACTTTGAAGGGTAC     750

FLO9_S288C        751 GTATATACTTTTGACAACAATCTAAGCCAGCCAAACTGTACCATTCCAGA     800
                      ||||||||||||||||||||||||||||||||||||||||||||||||||
FLO9_BY4741       751 GTATATACTTTTGACAACAATCTAAGCCAGCCAAACTGTACCATTCCAGA     800
```

```
FLO9_S288C        801 CCCTTCAAATTATACTGTCAGTACTACCATAACTACAACGGAACCATGGA    850
                      |||||||||||||||||||||||||||||||||||||||.||.|||||||
FLO9_BY4741       801 CCCTTCAAATTATACTGTCAGTACTACCATAACTACAACCGAGCCATGGA    850

FLO9_S288C        851 CCGGTACTTTCACTTCTACATCTACTGAAATGACCACCGTCACCGGTACC    900
                      |||||||||||||.|||||.||||||||.|||||.||..||||.||.|||
FLO9_BY4741       851 CCGGTACTTTCACCTCTACGTCTACTGAGATGACTACTATCACTGGCACC    900

FLO9_S288C        901 AACGGCGTTCCAACTGACGAAACCGTCATTGTCATCAGAACTCCAACAAC    950
                      |||||.||.|||||||||||||||.|||||||..|||.|||.||||||||
FLO9_BY4741       901 AACGGTGTACCAACTGACGAAACCATCATTGTTGTCAAAACACCAACAAC    950

FLO9_S288C        951 TGCTAGCACCATCATAACTACAACTGAGCCATGGAACAGCACTTTTACCT   1000
                      |||||||||||||||||||||.||.||.|||||||.|.|.|||||.||.|
FLO9_BY4741       951 TGCTAGCACCATCATAACTACGACCGAACCATGGACCGGTACTTTCACAT   1000

FLO9_S288C       1001 CTACTTCTACCGAATTGACCACAGTCACTGGCACCAATGGTGTACGAACT   1050
                      ||||.||.||.|||.||||.||..|||||||||||||.|||||||.||||
FLO9_BY4741      1001 CTACATCCACAGAAATGACTACTATCACTGGCACCAACGGTGTACCAACT   1050

FLO9_S288C       1051 GACGAAACCATCATTGTAATCAGAACACCAACAACAGCCACTACTGCCAT   1100
                      |||||||||||||||||..|||.||||||||||||.||.|..||...|||
FLO9_BY4741      1051 GACGAAACCATCATTGTTGTCAAAACACCAACAACTGCTAGCACCATCAT   1100

FLO9_S288C       1101 AACTACAACTGAGCCATGGAACAGCACTTTTACCTCTACTTCTACCGAAT   1150
                      ||||||||||||||||||||...||||||..||.||||||||||||||||
FLO9_BY4741      1101 AACTACAACTGAGCCATGGACTGGCACTTCCACTTCTACTTCTACCGAAT   1150

FLO9_S288C       1151 TGACCACAGTCACCGGTACCAATGGTTTGCCAACTGATGAGACCATCATT   1200
                      |||||||||||||||||||.|||||.|||||||||||.||.|||||||||
FLO9_BY4741      1151 TGACCACAGTCACCGGTACTAATGGCTTGCCAACTGACGAAACCATCATT   1200

FLO9_S288C       1201 GTCATCAGAACACCAACAACAGCCACTACTGCCATGACTACAACTCAGCC   1250
                      ||..||||||||||||||||.||.|..||...|||.|||||||||.||||
FLO9_BY4741      1201 GTTGTCAGAACACCAACAACTGCTAGCACCATCATAACTACAACTGAGCC   1250

FLO9_S288C       1251 ATGGAACGACACTTTTACCTCTACTTCTACCGAATTGACCACAGTCACCG   1300
                      |||||..|.|||||..||.|||||||||||||||||||||||||||||||
FLO9_BY4741      1251 ATGGACTGGCACTTCCACTTCTACTTCTACCGAATTGACCACAGTCACCG   1300

FLO9_S288C       1301 GTACCAATGGTTTGCCAACTGATGAGACCATCATTGTCATCAGAACACCA   1350
                      ||||||||||.||||||||||||||.|||||||||||..|||||||||||
FLO9_BY4741      1301 GTACCAATGGCTTGCCAACTGATGAAACCATCATTGTTGTCAGAACACCA   1350

FLO9_S288C       1351 ACAACAGCCACTACTGCCATGACTACAACTCAGCCATGGAACGACACTTT   1400
                      |||||.||.|..||...|||.|||||||||.|||||||||..|.|||||.
FLO9_BY4741      1351 ACAACTGCTAGCACCATCATAACTACAACTGAGCCATGGACTGGCACTTC   1400

FLO9_S288C       1401 TACCTCTACTTCTACCGAATTGACCACAGTCACCGGTACCAATGGTTTGC   1450
                      .||.|||||||||||||||||||||||||||||||||||||||||.||||
FLO9_BY4741      1401 CACTTCTACTTCTACCGAATTGACCACAGTCACCGGTACCAATGGCTTGC   1450

FLO9_S288C       1451 CAACTGATGAGACCATCATTGTCATCAGAACACCAACAACAGCCACTACT   1500
                      ||||||||||.|||||||||||..||||||||||||||||.||.|..||.
FLO9_BY4741      1451 CAACTGATGAAACCATCATTGTTGTCAGAACACCAACAACTGCTAACACC   1500

FLO9_S288C       1501 GCCATGACTACAACTCAGCCATGGAACGACACTTTTACCTCTACATCCAC   1550
                      ..|||.||||.||||.|||||||||..|.|||||..||.|||||.||.||
FLO9_BY4741      1501 ATCATAACTATAACTGAGCCATGGACTGGCACTTCCACTTCTACTTCTAC   1550

FLO9_S288C       1551 TGAAATCACCACCGTCACCGGTACCAATGGTTTGCCAACTGATGAGACCA   1600
                      .|||.|.|||||.|||||||||||||||||.||||||||||||||.||||
```

Biology Open • Supplementary information

```
FLO9_BY4741      1551 CGAATTGACCACAGTCACCGGTACCAATGGCTTGCCAACTGATGAAACCA   1600

FLO9_S288C       1601 TCATTGTCATCAGAACACCAACAACAGCCACTACTGCCATGACTACAACT   1650
                      |||||||..|||.||||||||||||.||.|..||...|||.|||||.||.
FLO9_BY4741      1601 TCATTGTTGTCAAAACACCAACAACTGCTAGCACCATCATAACTACGACC   1650

FLO9_S288C       1651 CAGCCATGGAACGACACTTTTACCTCTACATCCACTGAAATGACCACCGT   1700
                      .|.|||||||.||..|||||.||.|||||||||||.||||||||.||.||
FLO9_BY4741      1651 GAACCATGGACCGGTACTTTCACATCTACATCCACAGAAATGACTACTGT   1700

FLO9_S288C       1701 CACCGGTACCAACGGTTTGCCAACTGATGAAACCATCATTGTCATCAGAA   1750
                      |||.||||||||||||...||||||||||||||..|||||||..|.|.||
FLO9_BY4741      1701 CACTGGTACCAACGGTCAACCAACTGATGAAACTGTCATTGTTGTTAAAA   1750

FLO9_S288C       1751 CACCAACAACAGCCACTACTGCCATAACTACAACTGAGCCATGGAACAGC   1800
                      ||||.|||||.||.|..||...|||||||||||||||||||||||...||
FLO9_BY4741      1751 CACCTACAACTGCTAACACCATCATAACTACAACTGAGCCATGGACTGGC   1800

FLO9_S288C       1801 ACTTTTACCTCTACATCCACTGAAATGACCACCGTCACCGGTACCAACGG   1850
                      |||||.||||||||.||.|||||.|||||.||..||||.||.||||||||
FLO9_BY4741      1801 ACTTTCACCTCTACGTCTACTGAGATGACTACTATCACTGGCACCAACGG   1850

FLO9_S288C       1851 TTTGCCAACTGATGAAACCATCATTGTCATCAGAACACCAACAACAGCCA   1900
                      |.|.||||||||.||||||||||||||..|||.||||||||||||.||.|
FLO9_BY4741      1851 TGTACCAACTGACGAAACCATCATTGTTGTCAAAACACCAACAACTGCTA   1900

FLO9_S288C       1901 CTACTGCCATAACTACAACTCAGCCATGGAACGACACTTTTACCTCTACA   1950
                      ..||...|||||||||.||..|.|||||||.||..|||||.||.||||||
FLO9_BY4741      1901 GCACCATCATAACTACGACCGAACCATGGACCGGTACTTTCACATCTACA   1950

FLO9_S288C       1951 TCCACTGAAATGACCACCGTCACCGGTACCAACGGTTTGCCAACTGATGA   2000
                      |||||.||||||||.||..||||.||.|||||||||.|.||||||||.||
FLO9_BY4741      1951 TCCACAGAAATGACTACTATCACTGGCACCAACGGTGTACCAACTGACGA   2000

FLO9_S288C       2001 AACCATCATTGTCATCAGAACACCAACAACAGCCACTACTGCCATGACTA   2050
                      ||||||||||||..|||.||||||||||||.||.|..||...|||.||||
FLO9_BY4741      2001 AACCATCATTGTTGTCAAAACACCAACAACTGCTAGCACCATCATAACTA   2050

FLO9_S288C       2051 CAACTCAGCCATGGAACGACACTTTTACCTCTACATCCACTGAAATCACC   2100
                      |||||.|||||||||..|.||||||.||||||||.||.|||||.||.||.
FLO9_BY4741      2051 CAACTGAGCCATGGACTGGCACTTTCACCTCTACGTCTACTGAGATGACT   2100

FLO9_S288C       2101 ACCGTCACCGGTACCAACGGTTTGCCAACTGATGAGACCATCATTGTCAT   2150
                      ||..||||.||.|||||||||.|.||||||||.||.|||||||||||..|
FLO9_BY4741      2101 ACTATCACTGGCACCAACGGTGTACCAACTGACGAAACCATCATTGTTGT   2150

FLO9_S288C       2151 CAGAACACCAACAACAGCCACTACTGCCATGACTACAACTCAGCCATGGA   2200
                      ||.||||||||||||.||.|..||...|||.|||||.||..|.|||||||
FLO9_BY4741      2151 CAAAACACCAACAACTGCTAGCACCATCATAACTACGACCGAACCATGGA   2200

FLO9_S288C       2201 ACGACACTTTTACCTCTACATCCACTGAAATGACCACCGTCACCGGTACC   2250
                      .||..|||||.||.|||||||||||.||||||||.||.||||||||||||
FLO9_BY4741      2201 CCGGTACTTTCACATCTACATCCACAGAAATGACTACTGTCACCGGTACC   2250

FLO9_S288C       2251 AACGGCGTTCCAACTGACGAAACCGTCATTGTCATCAGAACTCCAACTAG   2300
                      |||||....||||||||||||||.||.|||||.||||||||.||||||||
FLO9_BY4741      2251 AACGGTCAACCAACTGACGAAACTGTGATTGTTATCAGAACCCCAACTAG   2300

FLO9_S288C       2301 TGAAGGTCTAATCAGCACCACCACTGAACCATGGACTGGTACTTTCACCT   2350
                      |||||||.|..|.|..|||||||||||||||||||||||||||||.||||
FLO9_BY4741      2301 TGAAGGTTTGGTTACAACCACCACTGAACCATGGACTGGTACTTTTACCT   2350

FLO9_S288C       2351 CTACATCCACTGAGATGACCACCGTCACCGGTACTAACGGTCAACCAACT   2400
```

```
                        |||||||.||||||||||||||||||||||||||.|||||||||||||||
FLO9_BY4741      2351 CTACATCTACTGAGATGACCACCGTCACCGGTACCAACGGTCAACCAACT   2400

FLO9_S288C       2401 GACGAAACCGTGATTGTTATCAGAACTCCAACCAGTGAAGGTTTGGTTAC   2450
                      ||||||||.|||||||||||||||||.|||||.|||||||||||||||||
FLO9_BY4741      2401 GACGAAACTGTGATTGTTATCAGAACCCCAACTAGTGAAGGTTTGGTTAC   2450

FLO9_S288C       2451 AACTACAACCGAGCCATGGACCGGTACTTTCACCTCTACATCTACTGAGA   2500
                      |||.||.||.||.||||||||.||||||||.|||||||||||||||||||
FLO9_BY4741      2451 AACCACCACTGAACCATGGACTGGTACTTTTACCTCTACATCTACTGAGA   2500

FLO9_S288C       2501 TGACCACCATCACTGGAACCAACGGTCAACCAACTGATGAAACTGTCATT   2550
                      ||||||||||||||||||||||||||||||||||||||||||||||||||
FLO9_BY4741      2501 TGACCACCATCACTGGAACCAACGGTCAACCAACTGATGAAACTGTCATT   2550

FLO9_S288C       2551 ATTGTCAAAACTCCAACTACTGCCATCTCATCCAGTTTGTCATCTTCTTC   2600
                      ||||||||||||||||||||||||||||||||||||||||||||||||||
FLO9_BY4741      2551 ATTGTCAAAACTCCAACTACTGCCATCTCATCCAGTTTGTCATCTTCTTC   2600

FLO9_S288C       2601 AGGACAAATCACCAGCTTTATCACGTCTGCGTCCAATTATTACCCCAT   2650
                      ||||||||||||||||||||||||||||.|||||||||||||||||||||
FLO9_BY4741      2601 AGGACAAATCACCAGCTTTATCACGTCTTCGCGTCCAATTATTACCCCAT   2650

FLO9_S288C       2651 TCTATCCTAGCAATGGAACTTCTGTGATTTCCTCCTCAGTAATTTCTTCC   2700
                      ||||||||||||||||||||||||||||||||||||||||||||||||||
FLO9_BY4741      2651 TCTATCCTAGCAATGGAACTTCTGTGATTTCCTCCTCAGTAATTTCTTCC   2700

FLO9_S288C       2701 TCAGACACTTCTTCTCTAGTCATTTCTTCCTCAGTCACTTCTTCTCTAGT   2750
                      ||||||||||||||||||||||||||||||||||||||||||||||||||
FLO9_BY4741      2701 TCAGACACTTCTTCTCTAGTCATTTCTTCCTCAGTCACTTCTTCTCTAGT   2750

FLO9_S288C       2751 CACTTCTTCTCCAGTCATTTCTTCTTCATTCATTTCTTCCCCTGTCATTT   2800
                      ||||||||||||||||||||||||||||||||||||||||||||||||||
FLO9_BY4741      2751 CACTTCTTCTCCAGTCATTTCTTCTTCATTCATTTCTTCCCCTGTCATTT   2800

FLO9_S288C       2801 CTTCTACAACAACCTCCGCTTCTATACTCTCTGAATCATCTAAATCATCC   2850
                      ||||||||||||||||||||||||||||||||||||||||||||||||||
FLO9_BY4741      2801 CTTCTACAACAACCTCCGCTTCTATACTCTCTGAATCATCTAAATCATCC   2850

FLO9_S288C       2851 GTCATTCCAACCAGTAGTTCCACCTCTGGTTCTTCTGAGAGCGAAACGGG   2900
                      ||||||||||||||||||||||||||||||||||||||||||||||||||
FLO9_BY4741      2851 GTCATTCCAACCAGTAGTTCCACCTCTGGTTCTTCTGAGAGCGAAACGGG   2900

FLO9_S288C       2901 TTCAGCTAGTTCTGCCTCTTCTTCCTCTTCTATCTCTTCTGAATCACCAA   2950
                      ||||||||||||||||||||||||||||||||||||||||||||||||||
FLO9_BY4741      2901 TTCAGCTAGTTCTGCCTCTTCTTCCTCTTCTATCTCTTCTGAATCACCAA   2950

FLO9_S288C       2951 AGTCTACATATTCGTCTTCATCATTACCACCTGTTACCAGTGCAACAACA   3000
                      ||||||||||||||||||||||||||||||||||||||||||||||||||
FLO9_BY4741      2951 AGTCTACATATTCGTCTTCATCATTACCACCTGTTACCAGTGCAACAACA   3000

FLO9_S288C       3001 AGTCAGGAAATTACTTCTTCATTACCACCTGTTACCACTACAAAAACGAG   3050
                      ||||||||||||||||||||||||||||||||||||||||||||||||||
FLO9_BY4741      3001 AGTCAGGAAATTACTTCTTCATTACCACCTGTTACCACTACAAAAACGAG   3050

FLO9_S288C       3051 CGAACAAACCACTTTGGTTACCGTGACATCCTGCGAATCTCATGTGTGCA   3100
                      ||||||||||||||||||||||||||||||||||||||||||||||||||
FLO9_BY4741      3051 CGAACAAACCACTTTGGTTACCGTGACATCCTGCGAATCTCATGTGTGCA   3100

FLO9_S288C       3101 CTGAATCTATCTCCTCTGCGATTGTTTCCACGGCCACCGTTACTGTTAGC   3150
                      ||||||||||||||||||||||||||||||||||||||||||||||||||
FLO9_BY4741      3101 CTGAATCTATCTCCTCTGCGATTGTTTCCACGGCCACCGTTACTGTTAGC   3150
```

Biology Open • Supplementary information

```
FLO9_S288C      3151 GGTGCCACAACAGAGTATACCACATGGTGCCCTATTTCTACCACAGAGAT   3200
                     ||||||||||||||||||||||||||||||||||||||||||||||||||
FLO9_BY4741     3151 GGTGCCACAACAGAGTATACCACATGGTGCCCTATTTCTACCACAGAGAT   3200

FLO9_S288C      3201 AACAAAGCAAACTACGGAGACAACAAAGCAAACCAAGGGGACAACAGAGC   3250
                     ||||||||||||||||||||||||||||||||||||||||||||||||||
FLO9_BY4741     3201 AACAAAGCAAACTACGGAGACAACAAAGCAAACCAAGGGGACAACAGAGC   3250

FLO9_S288C      3251 AAACCACAGAAACAACAAAACAAACCACAGTAGTTACAATTTCTTCTTGT   3300
                     ||||||||||||||||||||||||||||||||||||||||||||||||||
FLO9_BY4741     3251 AAACCACAGAAACAACAAAACAAACCACAGTAGTTACAATTTCTTCTTGT   3300

FLO9_S288C      3301 GAATCTGACGTATGCTCTAAGACTGCTTCTCCAGCCATTGTATCTACAAG   3350
                     ||||||||||||||||||||||||||||||||||||||||||||||||||
FLO9_BY4741     3301 GAATCTGACGTATGCTCTAAGACTGCTTCTCCAGCCATTGTATCTACAAG   3350

FLO9_S288C      3351 CACTGCTACTATTAATGGCGTTACCACAGAATACACAACATGGTGTCCTA   3400
                     ||||||||||||||||||||||||||||||||||||||||||||||||||
FLO9_BY4741     3351 CACTGCTACTATTAATGGCGTTACCACAGAATACACAACATGGTGTCCTA   3400

FLO9_S288C      3401 TTTCCACCACAGAATCGAAGCAACAAACTACGCTAGTTACTGTTACTTCC   3450
                     ||||||||||||||||||||||||||||||||||||||||||||||||||
FLO9_BY4741     3401 TTTCCACCACAGAATCGAAGCAACAAACTACGCTAGTTACTGTTACTTCC   3450

FLO9_S288C      3451 TGCGGATCTGGTGTGTGTTCCGAAACTACTTCACCTGCCATTGTTTCGAC   3500
                     ||||||||||||||||||||||||||||||||||||||||||||||||||
FLO9_BY4741     3451 TGCGGATCTGGTGTGTGTTCCGAAACTACTTCACCTGCCATTGTTTCGAC   3500

FLO9_S288C      3501 GGCCACGGCTACTGTGAATGATGTTGTTACGGTCTATTCTACATGGAGGC   3550
                     ||||||||||||||||||||||||||||||||||||||||||||||||||
FLO9_BY4741     3501 GGCCACGGCTACTGTGAATGATGTTGTTACGGTCTATTCTACATGGAGGC   3550

FLO9_S288C      3551 CACAGACTACGAATGAACAGTCTGTCAGCTCTAAAATGAACAGTGCTACC   3600
                     ||||||||||||||||||||||||||||||||||||||||||||||||||
FLO9_BY4741     3551 CACAGACTACGAATGAACAGTCTGTCAGCTCTAAAATGAACAGTGCTACC   3600

FLO9_S288C      3601 AGTGAGACAACAACCAATACTGGAGCTGCTGAGACAACTACCAGTACTGG   3650
                     ||||||||||||||||||||||||||||||||||||||||||||||||||
FLO9_BY4741     3601 AGTGAGACAACAACCAATACTGGAGCTGCTGAGACAACTACCAGTACTGG   3650

FLO9_S288C      3651 AGCTGCTGAGACGAAAACAGTAGTCACCTCTTCAATTTCAAGATTCAATC   3700
                     ||||||||||||||||||||||||||||||||||||||||||||||||||
FLO9_BY4741     3651 AGCTGCTGAGACGAAAACAGTAGTCACCTCTTCAATTTCAAGATTCAATC   3700

FLO9_S288C      3701 ATGCTGAAACACAGACGGCTTCCGCGACCGATGTGATTGGTCACAGCAGT   3750
                     ||||||||||||||||||||||||||||||||||||||||||||||||||
FLO9_BY4741     3701 ATGCTGAAACACAGACGGCTTCCGCGACCGATGTGATTGGTCACAGCAGT   3750

FLO9_S288C      3751 AGTGTTGTTTCTGTATCCGAAACTGGCAACACCAAGAGTCTAACAAGTTC   3800
                     ||||||||||||||||||||||||||||||||||||||||||||||||||
FLO9_BY4741     3751 AGTGTTGTTTCTGTATCCGAAACTGGCAACACCAAGAGTCTAACAAGTTC   3800

FLO9_S288C      3801 CGGGTTGAGTACTATGTCGCAACAGCCTCGTAGCACACCAGCAAGTAGCA   3850
                     ||||||||||||||||||||||||||||||||||||||||||||||||||
FLO9_BY4741     3801 CGGGTTGAGTACTATGTCGCAACAGCCTCGTAGCACACCAGCAAGTAGCA   3850

FLO9_S288C      3851 TGGTAGGATCTAGTACAGCTTCTTTAGAAATTTCAACGTATGCTGGCAGT   3900
                     ||||||||||||||||||||||||||||||||||||||||||||||||||
FLO9_BY4741     3851 TGGTAGGATCTAGTACAGCTTCTTTAGAAATTTCAACGTATGCTGGCAGT   3900

FLO9_S288C      3901 GCCAACAGCTTACTGGCCGGTAGTGGTTTAAGTGTCTTCATTGCGTCCTT   3950
                     ||||||||||||||||||||||||||||||||||||||||||||||||||
FLO9_BY4741     3901 GCCAACAGCTTACTGGCCGGTAGTGGTTTAAGTGTCTTCATTGCGTCCTT   3950
```

```
FLO9_S288C       3951 ATTGCTGGCAATTATTTAA   3969
                      |||||||||||||||||||
FLO9_BY4741      3951 ATTGCTGGCAATTATTTAA   3969
```

Fig. S4. Nucleotide sequence alignment of *FLO9* (*S. cerevisiae* S288C vs. BY4741) with 92.9% identity.

Biology Open • Supplementary information

```
Flo9p_S288C        1 MSLAHYCLLLAIVTLLGLTNVVSATTAACLPANSRKNGMNVNFYQYSLRD     50
                     ||||||||||||||||||||||||||||||||||||||||||||||||||
Flo9p_BY4741       1 MSLAHYCLLLAIVTLLGLTNVVSATTAACLPANSRKNGMNVNFYQYSLRD     50

Flo9p_S288C       51 SSTYSNAAYMAYGYASKTKLGSVGGQTDISIDYNIPCVSSSGTFPCPQED    100
                     ||||||||||||||||||||||||||||||||||||||||||||||||||
Flo9p_BY4741      51 SSTYSNAAYMAYGYASKTKLGSVGGQTDISIDYNIPCVSSSGTFPCPQED    100

Flo9p_S288C      101 LYGNWGCKGIGACSNNPIIAYWSTDLFGFYTTPTNVTLEMTGYFLPPQTG    150
                     ||||||||||||||||||||||||||||||||||||||||||||||||||
Flo9p_BY4741     101 LYGNWGCKGIGACSNNPIIAYWSTDLFGFYTTPTNVTLEMTGYFLPPQTG    150

Flo9p_S288C      151 SYTFKFATVDDSAILSVGGSIAFECCAQEQPPITSTNFTINGIKPWNGSP    200
                     ||||||||||||||||||||||||||||||||||||||||||||||||||
Flo9p_BY4741     151 SYTFKFATVDDSAILSVGGSIAFECCAQEQPPITSTNFTINGIKPWNGSP    200

Flo9p_S288C      201 PDNITGTVYMYAGFYYPMKIVYSNAVAWGTLPISVTLPDGTTVSDDFEGY    250
                     ||||||||||||||||||||||||||||||||||||||||||||||||||
Flo9p_BY4741     201 PDNITGTVYMYAGFYYPMKIVYSNAVAWGTLPISVTLPDGTTVSDDFEGY    250

Flo9p_S288C      251 VYTFDNNLSQPNCTIPDPSNYTVSTTITTTEPWTGTFTSTSTEMTTVTGT    300
                     ||||||||||||||||||||||||||||||||||||||||||||||:|||
Flo9p_BY4741     251 VYTFDNNLSQPNCTIPDPSNYTVSTTITTTEPWTGTFTSTSTEMTTITGT    300

Flo9p_S288C      301 NGVPTDETVIVIRTPTTASTIITTTEPWNSTFTSTSTELTTVTGTNGVRT    350
                     ||||||||:||::|||||||||||||||..||||||||:||:||||||.|
Flo9p_BY4741     301 NGVPTDETIIVVKTPTTASTIITTTEPWTGTFTSTSTEMTTITGTNGVPT    350

Flo9p_S288C      351 DETIIVIRTPTTATTAITTTEPWNSTFTSTSTELTTVTGTNGLPTDETII    400
                     ||||||::|||||:|.|||||||..|.|||||||||||||||||||||||
Flo9p_BY4741     351 DETIIVVKTPTTASTIITTTEPWTGTSTSTSTELTTVTGTNGLPTDETII    400

Flo9p_S288C      401 VIRTPTTATTAMTTTQPWNDTFTSTSTELTTVTGTNGLPTDETIIVIRTP    450
                     |:||||||:|.:|||:||..|.||||||||||||||||||||||||:|||
Flo9p_BY4741     401 VVRTPTTASTIITTTEPWTGTSTSTSTELTTVTGTNGLPTDETIIVVRTP    450

Flo9p_S288C      451 TTATTAMTTTQPWNDTFTSTSTELTTVTGTNGLPTDETIIVIRTPTTATT    500
                     |||:|.:|||:||..|.||||||||||||||||||||||||:||||||.|
Flo9p_BY4741     451 TTASTIITTTEPWTGTSTSTSTELTTVTGTNGLPTDETIIVVRTPTTANT    500

Flo9p_S288C      501 AMTTTQPWNDTFTSTSTEITTVTGTNGLPTDETIIVIRTPTTATTAMTTT    550
                     .:|.|:||..|.||||||:|||||||||||||||||::|||||:|.:|||
Flo9p_BY4741     501 IITITEPWTGTSTSTSTELTTVTGTNGLPTDETIIVVKTPTTASTIITTT    550

Flo9p_S288C      551 QPWNDTFTSTSTEMTTVTGTNGLPTDETIIVIRTPTTATTAITTTEPWNS    600
                     :||..|||||||||||||||||.|||||:||::|||||.|.|||||||..
Flo9p_BY4741     551 EPWTGTFTSTSTEMTTVTGTNGQPTDETVIVVKTPTTANTIITTTEPWTG    600

Flo9p_S288C      601 TFTSTSTEMTTVTGTNGLPTDETIIVIRTPTTATTAITTTQPWNDTFTST    650
                     |||||||||||:|||||:||||||||::|||||:|.||||:||..|||||
Flo9p_BY4741     601 TFTSTSTEMTTITGTNGVPTDETIIVVKTPTTASTIITTTEPWTGTFTST    650

Flo9p_S288C      651 STEMTTVTGTNGLPTDETIIVIRTPTTATTAMTTTQPWNDTFTSTSTEIT    700
                     ||||||:|||||:||||||||::|||||:|.:|||:||..||||||||:|
Flo9p_BY4741     651 STEMTTITGTNGVPTDETIIVVKTPTTASTIITTTEPWTGTFTSTSTEMT    700

Flo9p_S288C      701 TVTGTNGLPTDETIIVIRTPTTATTAMTTTQPWNDTFTSTSTEMTTVTGT    750
                     |:|||||:||||||||::|||||:|.:|||:||..|||||||||||||||
Flo9p_BY4741     701 TITGTNGVPTDETIIVVKTPTTASTIITTTEPWTGTFTSTSTEMTTVTGT    750

Flo9p_S288C      751 NGVPTDETVIVIRTPTSEGLISTTTEPWTGTFTSTSTEMTTVTGTNGQPT    800
                     ||.|||||||||||||||||::||||||||||||||||||||||||||||
Flo9p_BY4741     751 NGQPTDETVIVIRTPTSEGLVTTTTEPWTGTFTSTSTEMTTVTGTNGQPT    800
```

Biology Open • Supplementary information

```
Flo9p_S288C      801 DETVIVIRTPTSEGLVTTTTEPWTGTFTSTSTEMTTITGTNGQPTDETVI    850
                     ||||||||||||||||||||||||||||||||||||||||||||||||||
Flo9p_BY4741     801 DETVIVIRTPTSEGLVTTTTEPWTGTFTSTSTEMTTITGTNGQPTDETVI    850

Flo9p_S288C      851 IVKTPTTAISSSLSSSSGQITSFITSARPIITPFYPSNGTSVISSSVISS    900
                     ||||||||||||||||||||||||||:|||||||||||||||||||||||
Flo9p_BY4741     851 IVKTPTTAISSSLSSSSGQITSFITSSRPIITPFYPSNGTSVISSSVISS    900

Flo9p_S288C      901 SDTSSLVISSSVTSSLVTSSPVISSSFISSPVISSTTTSASILSESSKSS    950
                     ||||||||||||||||||||||||||||||||||||||||||||||||||
Flo9p_BY4741     901 SDTSSLVISSSVTSSLVTSSPVISSSFISSPVISSTTTSASILSESSKSS    950

Flo9p_S288C      951 VIPTSSSTSGSSESETGSASSASSSSSISSESPKSTYSSSSLPPVTSATT   1000
                     ||||||||||||||||||||||||||||||||||||||||||||||||||
Flo9p_BY4741     951 VIPTSSSTSGSSESETGSASSASSSSSISSESPKSTYSSSSLPPVTSATT   1000

Flo9p_S288C     1001 SQEITSSLPPVTTTKTSEQTTLVTVTSCESHVCTESISSAIVSTATVTVS   1050
                     ||||||||||||||||||||||||||||||||||||||||||||||||||
Flo9p_BY4741    1001 SQEITSSLPPVTTTKTSEQTTLVTVTSCESHVCTESISSAIVSTATVTVS   1050

Flo9p_S288C     1051 GATTEYTTWCPISTTEITKQTTETTKQTKGTTEQTTETTKQTTVVTISSC   1100
                     ||||||||||||||||||||||||||||||||||||||||||||||||||
Flo9p_BY4741    1051 GATTEYTTWCPISTTEITKQTTETTKQTKGTTEQTTETTKQTTVVTISSC   1100

Flo9p_S288C     1101 ESDVCSKTASPAIVSTSTATINGVTTEYTTWCPISTTESKQQTTLVTVTS   1150
                     ||||||||||||||||||||||||||||||||||||||||||||||||||
Flo9p_BY4741    1101 ESDVCSKTASPAIVSTSTATINGVTTEYTTWCPISTTESKQQTTLVTVTS   1150

Flo9p_S288C     1151 CGSGVCSETTSPAIVSTATATVNDVVTVYSTWRPQTTNEQSVSSKMNSAT   1200
                     ||||||||||||||||||||||||||||||||||||||||||||||||||
Flo9p_BY4741    1151 CGSGVCSETTSPAIVSTATATVNDVVTVYSTWRPQTTNEQSVSSKMNSAT   1200

Flo9p_S288C     1201 SETTTNTGAAETTTSTGAAETKTVVTSSISRFNHAETQTASATDVIGHSS   1250
                     ||||||||||||||||||||||||||||||||||||||||||||||||||
Flo9p_BY4741    1201 SETTTNTGAAETTTSTGAAETKTVVTSSISRFNHAETQTASATDVIGHSS   1250

Flo9p_S288C     1251 SVVSVSETGNTKSLTSSGLSTMSQQPRSTPASSMVGSSTASLEISTYAGS   1300
                     ||||||||||||||||||||||||||||||||||||||||||||||||||
Flo9p_BY4741    1251 SVVSVSETGNTKSLTSSGLSTMSQQPRSTPASSMVGSSTASLEISTYAGS   1300

Flo9p_S288C     1301 ANSLLAGSGLSVFIASLLLAII*   1323
                     ||||||||||||||||||||||
Flo9p_BY4741    1301 ANSLLAGSGLSVFIASLLLAII*   1323
```

Fig. S5. Amino acid sequence alignment of Flo9p (*S. cerevisiae* S288C vs. BY4741) with 93% identity.

Table S1. Primers used in this study

Primer	Sequence	Description	Used with	Expected size of PCR product
**Purpose: For preparing *FLO9* insert**<br>**DNA template: genomic DNA isolated from a SWORD clone**				
CN0084	5'- CCCAAGCTTATGTCTCTGGCACATTATTG TTTAC -3'	Forward (*Hin*dIII recognition site - underlined)	CN0085	3,987 bp
CN0085	5'- TTTCCCGGGTTAAATAATTGCCAGCAATA AGGACG -3'	Reverse (*Sma*I recognition site - underlined)	CN0084	3,987 bp
**Purpose: For preparing DNA cassette (*FLO9* from SWORD and hygromycin resistance gene)**<br>**DNA template: Ligation reaction of pFA6a-hphMX6 and *FLO9* insert**				
CN0087	5'- ATGTCTCTGGCACATTATTG -3'	Forward; Located at nt1-20 of *FLO9* coding sequence	CN0060	5,718 bp
CN0060	5'- TATTTAGCAAAGAAAAGATACACAGATAC GTAAAAAGAACGCGAATTTTAATCGATGA ATTCGAGCTCG -3'	Reverse: 50 bp downstream of *FLO9* stop codon + homology sequence to pFA6a-hphMX6 (underlined)	CN0087	5,718 bp
**Purpose: For yeast strain verification (sending out PCR products for DNA sequencing)**<br>**DNA template: genomic DNA isolated from yeast *ALD4::GFP* (reference clone) transformed with DNA cassette (*FLO9* from SWORD and hygromycin resistance gene)**				
CN0086	5'- CCTGCCAAATTATTCTACCTTC -3'	Forward; Located at 150 nt upstream of *FLO9* start codon	CN0096	4,455 bp
CN0096	5'- CTCAGTGGCAAATCCTAAC -3'	Reverse; Located at nt251-269 of hphMX6 sequence	CN0086	4,455 bp
**Sequencing primers**				
CN0086	5'- CCTGCCAAATTATTCTACCTTC -3'	Forward; Located at 150 nt upstream of *FLO9* start codon		
CN0087	5'- ATGTCTCTGGCACATTATTG – 3'	Forward; Located at nt1-20 of *FLO9* coding sequence		
CN0088	5'- CCTGATAATATTACAGGGACTG -3'	Forward; Located at nt601-622 of *FLO9* coding sequence		
CN0089	5'- GTGTGACACTACCAGATG -3'	Forward; Located at nt701-718 of *FLO9* coding sequence		
CN0091	5'- GACGAAACCGTGATTGTTATC - 3'	Forward; Located at nt2,401-2,421 of *FLO9* coding sequence		
CN0092	5'- TGTTGTTGCACTGGTAACAG -3'	Reverse; Located at nt2,981-3,000 of *FLO9* coding sequence		
CN0093	5'- AGTCAGGAAATTACTTCTTCATTACC -3'	Forward; Located at nt3,001-3,026 of *FLO9* coding sequence		
CN0094	5'- GGTAGCACTGTTCATTTTAG -3'	Reverse; Located at nt3,581-3,600 of *FLO9* coding sequence		
CN0095	5'- AGTGAGACAACAACCAATACTG -3'	Forward; Located at nt3,601-3,622 of *FLO9* coding sequence		

Table S2. Ald4p protein levels (at log-phase, saturation, and stationary phase) of SWORD clones, relative to those of *ALD4::GFP* reference clones

Log phase cultures		Band intensity (arbitrary unit)					
		ALD4::GFP (SWORD)			*ALD4::GFP* (Reference)		
		**Clone 4**	**Clone 5**	**Clone 8**	**Clone 2**	**Clone 3**	**Clone 5**
**Exp 1**	Anti-GFP	3975.15	9150.75	11642.92	7010.68	7992.90	12901.41
	Anti-alpha tubulin	19399.84	24827.79	26761.64	21880.92	20259.09	18538.62
	Ratio	**0.20**	**0.37**	**0.44**	**0.32**	**0.39**	**0.70**
**Exp 2**	Anti-GFP	13530.47	8382.67	14822.37	12867.14	9172.92	10457.30
	Anti-alpha tubulin	32532.69	31128.28	30483.66	33137.61	31329.20	28796.89
	Ratio	**0.42**	**0.27**	**0.49**	**0.39**	**0.29**	**0.36**
**Exp 3**	Anti-GFP	13968.75	5537.02	10502.65	5849.17	9811.33	8300.82
	Anti-alpha tubulin	14915.81	14645.92	11944.58	9353.04	9226.38	16289.11
	Ratio	**0.94**	**0.38**	**0.88**	**0.63**	**1.06**	**0.51**
**Average**		**0.52**	**0.34**	**0.60**	**0.44**	**0.58**	**0.52**
**SEM**		**0.22**	**0.03**	**0.14**	**0.09**	**0.24**	**0.10**
**Average of 3 clones**		**0.49**			**0.52**		
**Fold-change**		**0.94**			**1.00**		
**1-day cultures**		**Band intensity (arbitrary unit)**					
		ALD4::GFP (SWORD)			*ALD4::GFP* (Reference)		
		**Clone 4**	**Clone 5**	**Clone 8**	**Clone 2**	**Clone 3**	**Clone 5**
**Exp 1**	Anti-GFP	12341.61	11275.16	13743.15	16780.84	15424.58	14595.81
	Anti-alpha tubulin	27084.10	23569.22	30650.14	26158.41	25900.98	27394.46
	Ratio	**0.46**	**0.48**	**0.45**	**0.64**	**0.60**	**0.53**
**Exp 2**	Anti-GFP	6234.09	11348.71	8964.61	18024.44	11983.00	14458.33
	Anti-alpha tubulin	35462.45	31037.64	35469.59	31746.72	30499.03	26999.53
	Ratio	**0.18**	**0.37**	**0.25**	**0.57**	**0.39**	**0.54**
**Exp 3**	Anti-GFP	2292.28	7960.19	6397.58	5714.46	19303.23	19835.74
	Anti-alpha tubulin	18421.99	19429.64	21366.52	20039.74	22986.99	22076.94
	Ratio	**0.12**	**0.41**	**0.30**	**0.29**	**0.84**	**0.90**
**Average**		**0.25**	**0.42**	**0.33**	**0.50**	**0.61**	**0.66**
**SEM**		**0.10**	**0.03**	**0.06**	**0.11**	**0.13**	**0.12**
**Average of 3 Clones**		**0.33**			**0.59**		
**Fold-change**		**0.57**			**1.00**		
**5-day cultures**		**Band intensity (arbitrary unit)**					
		ALD4::GFP (SWORD)			*ALD4::GFP* (Reference)		
		**Clone 4**	**Clone 5**	**Clone 8**	**Clone 2**	**Clone 3**	**Clone 5**
**Exp 1**	Anti-GFP	2364.81	2297.71	4289.46	13216.21	7598.95	13046.50
	Anti-alpha tubulin	18028.79	19048.82	19931.45	20540.67	13817.02	23086.28
	Ratio	**0.13**	**0.12**	**0.22**	**0.64**	**0.55**	**0.57**
**Exp 2**	Anti-GFP	6649.25	10685.11	11315.76	20920.73	22727.96	18072.48

Biology Open • Supplementary information

	Anti-alpha tubulin	35460.33	30972.01	36950.23	34665.65	28654.48	29530.22
	Ratio	**0.19**	**0.34**	**0.31**	**0.60**	**0.79**	**0.61**
**Exp 3**	Anti-GFP	9761.11	7176.46	9708.48	10967.09	18613.08	11836.54
	Anti-alpha tubulin	16059.68	18021.02	18234.10	17890.00	16744.49	22316.49
	Ratio	**0.61**	**0.40**	**0.53**	**0.61**	**1.11**	**0.53**
**Average**		**0.31**	**0.29**	**0.35**	**0.62**	**0.82**	**0.57**
**SEM**		**0.15**	**0.09**	**0.09**	**0.01**	**0.16**	**0.02**
**Average of 3 Clones**		**0.32**			**0.67**		
**Fold-change**		**0.47**			**1.00**		

Biology Open • Supplementary information

Table S3. Raw data of length distribution analysis of Ald4p-GFP structures

Structure no.	Length (µm) of Ald4p-GFP structures		
	Reference	Reference with *FLO9* from SWORD	SWORD
1	0.706	0.434	0.288
2	0.279	0.446	4.499
3	0.487	0.238	0.981
4	0.307	0.971	0.334
5	0.784	1.113	0.206
6	0.633	0.527	0.211
7	0.578	0.453	0.197
8	0.231	1.445	0.240
9	0.439	0.407	0.181
10	0.344	0.471	0.273
11	0.269	0.739	1.602
12	1.257	0.570	1.449
13	0.521	0.240	1.084
14	0.344	1.921	1.924
15	0.181	0.501	2.200
16	0.140	0.475	0.330
17	0.547	0.189	0.176
18	0.566	1.541	0.627
19	0.641	0.351	0.156
20	0.700	0.865	0.185
21	0.309	0.452	0.868
22	0.670	0.203	1.123
23	0.340	0.907	0.199
24	0.834	1.050	0.902
25	0.521	0.372	1.361
26	0.692	1.861	3.303
27	0.620	0.900	3.392
28	0.977	1.000	0.758
29	0.275	0.397	1.297
30	0.713	1.825	2.125
31	0.241	0.702	0.215
32	0.268	0.741	0.186
33	0.189	1.852	1.684
34	0.968	0.328	0.982
35	0.250	0.312	0.243
36	0.160	1.571	1.873
37	0.949	1.781	2.480
38	0.723	1.161	1.170

Structure no.	Length (μm) of Ald4p-GFP structures		
	Reference	Reference with *FLO9* from SWORD	SWORD
39	0.226	0.411	0.170
40	0.680	0.448	0.241
41	0.262	0.225	1.392
42	0.240	1.489	0.149
43	0.226	0.546	1.780
44	0.279	0.605	2.722
45	1.199	1.479	1.099
46	0.278	0.645	0.258
47	0.271	1.558	2.352
48	0.587	1.033	0.799
49	0.591	0.262	0.940
50	0.844	0.304	3.208
51	0.188	1.151	2.095
52	0.397	1.326	2.555
53	0.227	0.383	2.137
54	0.246	0.343	0.165
55	0.519	0.283	1.011
56	0.877	0.638	0.870
57	0.447	0.656	3.075
58	0.719	0.399	0.127
59	0.208	2.649	0.552
60	0.245	1.175	0.253
61	0.821	0.647	0.159
62	0.860	0.722	1.926
63	1.655	1.689	3.082
64	1.400	1.057	0.718
65	0.467	0.610	3.423
66	1.575	0.535	0.257
67	1.096	1.454	0.254
68	0.225	0.969	0.229
69	0.185	0.456	2.382
70	0.305	0.383	2.298
71	0.760	1.988	0.367
72	0.279	1.276	0.232
73	0.167	0.952	0.170
74	0.509	0.239	0.174
75	0.296	1.170	0.241
76	1.070	0.398	0.252
77	0.393	0.170	0.179
78	1.243	2.088	0.732

Structure no.	Length (µm) of Ald4p-GFP structures		
	Reference	Reference with *FLO9* from SWORD	SWORD
79	0.210	0.367	0.240
80	0.331	0.762	1.156
81	0.420	0.350	2.455
82	0.299	1.157	0.996
83	0.197	0.419	1.875
84	1.056	1.589	0.149
85	0.118	0.283	2.128
86	0.169	0.345	0.167
87	1.000	0.282	0.186
88	0.569	1.048	1.814
89	0.570	0.815	0.279
90	1.843	1.232	0.256
91	0.484	1.695	0.344
92	0.253	1.814	0.176
93	1.098	1.489	0.208
94	0.450	0.215	0.205
95	0.133	0.329	1.526
96	0.280	0.347	1.925
97	0.281	0.832	3.045
98	0.282	0.721	0.182
99	0.689	0.632	0.720
100	0.234	1.963	0.256
101	0.470	0.350	1.402
102	1.167	0.600	0.457
103	0.260	0.200	0.890
104	0.287	0.598	0.259
105	0.882	0.876	1.629
106	0.334	1.125	0.216
107	0.752	0.740	1.042
108	0.277	1.147	1.503
109	0.228	0.223	0.815
110	0.411	0.609	0.442
111	0.151	1.079	2.149
112	0.251	0.569	0.247
113	0.181	0.313	0.873
114	0.158	0.708	0.209
115	1.201	0.735	1.759
116	0.222	0.184	1.176
117	0.322	1.885	0.371
118	0.328	0.366	0.780

Structure no.	Length (µm) of Ald4p-GFP structures		
	Reference	Reference with *FLO9* from SWORD	SWORD
119	0.960	1.639	3.292
120	0.948	0.427	1.369
121	0.400	1.177	2.169
122	0.270	1.171	0.146
123	0.507	1.678	0.715
124	1.127	0.610	0.246
125	0.226	0.457	1.937
126	1.006	0.856	3.660
127	0.198	0.176	1.914
128	0.998	0.621	0.954
129	1.453	0.685	1.267
130	0.795	1.638	0.216
131	0.617	1.401	0.918
132	0.181	0.403	1.148
133	0.393	0.364	0.303
134	0.548	0.806	0.259
135	0.512	1.553	0.449
136	0.899	2.010	0.785
137	1.633	0.480	2.786
138	0.535	0.516	0.196
139	0.917	1.254	0.686
140	0.693	0.242	0.181
141	0.175	0.475	0.482
142	0.633	1.423	1.236
143	0.616	0.878	2.749
144	0.815	1.562	0.828
145	0.889	0.710	0.242
146	0.188	0.297	2.422
147	0.194	1.009	1.847
148	0.602	1.539	1.935
149	0.811	0.987	1.559
150	0.711	0.237	0.363
151	0.812	0.516	0.156
152	0.416	0.912	0.931
153	0.468	0.746	2.738
154	0.556	0.503	0.189
155	1.726	0.646	0.377
156	0.551	0.812	1.026
157	1.312	2.301	1.005
158	0.441	1.153	1.100

Biology Open • Supplementary information

Structure no.	Length (µm) of Ald4p-GFP structures		
	Reference	Reference with *FLO9* from SWORD	SWORD
159	0.688	0.901	2.163
160	0.951	0.205	0.169
161	0.842	0.754	0.215
162	0.481	0.309	1.007
163	0.343	1.512	0.216
164	0.303	0.754	2.897
165	0.475	0.567	0.668
166	0.773	0.524	2.423
167	0.331	0.470	2.161
168	0.515	0.568	0.566
169	0.965	1.122	0.266
170	0.811	0.492	0.162
171	0.470	0.403	0.143
172	0.203	0.674	0.147
173	0.527	0.758	2.982
174	0.285	0.314	1.329
175	1.313	1.037	0.251
176	0.669	0.684	1.720
177	0.578	0.695	0.256
178	0.256	1.802	2.082
179	0.402	2.144	0.604
180	0.260	1.537	0.196
181	0.433	1.649	0.652
182	0.180	2.285	0.398
183	0.159	0.949	0.986
184	0.634	1.010	1.460
185	0.765	1.200	0.151
186	0.758	1.338	0.783
187	0.398	1.662	0.944
188	0.503	0.686	0.703
189	1.226	2.388	1.951
190	1.092	0.516	0.881
191	0.827	1.533	1.426
192	1.111	0.243	2.307
193	0.842	0.886	3.043
194	0.903	0.290	3.370
195	0.619	1.194	1.299
196	0.446	2.520	0.188
197	0.585	0.180	0.952
198	1.203	0.515	2.727

Structure no.	Length (μm) of Ald4p-GFP structures		
	Reference	Reference with *FLO9* from SWORD	SWORD
199	0.199	1.069	3.337
200	0.804	0.322	0.205
201	0.191	0.644	0.212
202	0.622	1.884	0.323
203	1.069	0.508	0.274
204	0.259	0.252	0.273
205	0.255	0.583	0.517
206	0.239	0.143	0.182
207	0.610	0.232	2.216
208	0.220	0.290	0.221
209	0.273	0.511	0.896
210	0.366	0.212	1.581
211	1.021	0.316	0.706
212	0.213	0.184	0.210
213	0.500	0.728	0.141
214	0.875	1.074	5.873
215	0.143	0.626	0.200
216	0.526	0.526	0.231
217	0.308	1.102	1.055
218	0.633	1.227	0.903
219	0.864	1.056	3.029
220	0.527	0.398	0.596
221	0.210	1.022	1.812
222	0.647	1.236	2.084
223	0.267	1.566	0.180
224	0.290	1.283	2.509
225	0.454	0.534	2.521
226	0.726	0.585	3.163
227	0.399	0.366	2.366
228	0.429	0.391	1.278
229	0.143	2.161	1.117
230	0.399	1.606	2.149
231	1.230	0.465	0.424
232	0.912	0.465	0.229
233	1.076	0.199	0.195
234	1.098	0.508	1.017
235	0.429	1.120	0.498
236	0.166	0.568	1.434
237	0.485	0.864	0.623
238	0.339	0.762	1.293

Structure no.	Length (µm) of Ald4p-GFP structures		
	Reference	Reference with *FLO9* from SWORD	SWORD
239	0.635	1.203	0.750
240	1.096	1.198	4.558
241	0.746	0.827	0.799
242	0.444	1.000	0.536
243	0.252	1.036	1.898
244	0.329	0.749	0.777
245	0.254	0.632	1.610
246	0.224	0.362	1.680
247	0.739	0.257	2.016
248	1.076	0.481	3.075
249	1.142	0.776	1.303
250	0.994	0.902	4.180
251	0.925	0.213	1.535
252	0.862	0.967	0.619
253	0.197	1.904	1.345
254	0.359	0.906	3.238
255	0.243	1.694	1.435
256	0.238	0.226	1.263
257	0.635	0.358	1.674
258	0.205	2.480	0.146
259	0.779	0.559	2.057
260	0.626	0.260	3.050
261	0.274	0.747	0.756
262	1.365	0.313	0.653
263	0.932	1.055	0.377
264	0.334	0.350	4.254
265	0.791	0.806	1.183
266	0.633	0.472	0.809
267	0.508	0.927	0.206
268	0.597	1.496	2.186
269	0.707	0.431	3.050
270	0.481	0.959	0.554
271	0.211	1.487	0.157
272	0.252	1.139	1.515
273	0.979	0.611	1.994
274	0.312	0.639	0.459
275	0.158	1.833	1.288
276	0.828	1.322	0.412
277	0.482	0.497	0.837
278	0.781	0.807	1.685

Structure no.	Length (μm) of Ald4p-GFP structures		
	Reference	Reference with *FLO9* from SWORD	SWORD
279	0.763	2.094	0.384
280	0.686	0.469	0.180
281	0.544	1.124	1.170
282	1.092	0.143	0.140
283	0.241	0.326	0.211
284	0.472	0.321	0.765
285	0.613	0.352	3.107
286	0.908	0.343	4.126
287	0.315	0.569	0.889
288	0.199	1.682	2.001
289	0.464	2.135	0.457
290	0.445	0.934	0.140
291	1.061	1.075	1.552
292	0.782	0.597	0.244
293	0.457	1.077	1.809
294	0.287	0.352	1.867
295	1.212	1.785	0.266
296	0.252	1.331	0.143
297	0.922	1.765	1.069
298	0.358	1.415	0.742
299	0.515	1.129	0.855
300	0.560	1.106	0.143
301	0.552	0.856	0.418
302	0.442	0.985	0.325
303	0.223	2.300	0.197
304	1.404	1.809	0.646
305	0.460	0.143	0.832
306	1.085	0.509	0.541
307	0.890	0.613	1.230
308	0.228	2.939	0.247
309	0.424	0.515	1.233
310	0.264	1.269	0.388
311	0.569	0.403	2.455
312	0.274	0.537	0.242
313	0.225	0.509	0.115
314	0.995	0.913	3.885
315	0.278	0.583	1.601
316	0.330	0.530	1.405
317	0.351	1.264	0.274
318	0.187	1.893	3.182

Structure no.	Length (μm) of Ald4p-GFP structures		
	Reference	Reference with *FLO9* from SWORD	SWORD
319	0.417	1.069	0.511
320	0.293	1.053	0.602
321	0.180	0.547	1.429
322	1.302	0.143	0.821
323	0.264	0.479	0.143
324	0.564	0.621	0.461
325	0.420	0.867	0.759
326	0.237	0.409	0.502
327	0.421	0.616	0.860
328	0.222	2.805	0.320
329	0.379	0.838	0.428
330	1.165	1.603	2.336
331	0.514	1.172	0.402
332	0.588	0.518	2.335
333	0.280	1.684	0.327
334	0.468	1.154	4.550
335	0.178	2.111	0.519
336	0.542	0.489	3.497
337	1.163	1.874	0.143
338	0.161	0.740	0.154
339	0.970	0.677	0.312
340	0.178	0.322	3.243
341	0.403	0.806	0.245
342	0.335	0.548	1.572
343	0.862	0.389	1.927
344	1.031	2.794	0.227
345	0.234	0.585	2.179
346	1.660	1.537	0.550
347	0.261	0.822	1.903
348	2.367	1.033	0.885
349	0.237	1.110	0.255
350	0.181	0.531	1.601
351	0.782	0.891	0.601
352	0.870	0.205	0.345
353	0.920	0.219	0.259
354	0.545	1.902	0.924
355	0.140	1.479	0.424
356	0.451	0.390	4.225
357	0.353	0.441	1.296
358	0.383	1.396	1.128

Biology Open • Supplementary information

Structure no.	Length (µm) of Ald4p-GFP structures		
	Reference	Reference with *FLO9* from SWORD	SWORD
359	0.204	0.202	2.465
360	1.767	0.292	2.533
361	0.218	0.366	2.367
362	0.242	0.806	2.750
363	0.191	0.803	0.273
364	0.255	1.102	1.082
365	0.324	1.031	0.993
366	0.280	0.613	4.037
367	0.430	0.427	2.861
368	0.661	0.713	0.892
369	0.659	0.617	0.341
370	0.419	0.506	2.946
371	0.721	1.082	3.273
372	0.607	0.501	0.909
373	0.426	1.868	0.704
374	0.935	0.962	1.100
375	0.866	0.399	1.383
376	0.481	0.415	1.383
377	0.927	0.213	0.171
378	0.544	0.663	1.807
379	0.679	0.920	2.340
380	0.834	0.194	0.194
381	1.087	1.097	1.197
382	0.386	1.075	0.513
383	0.390	1.412	1.453
384	0.480	0.379	0.859
385	0.573	0.681	0.199
386	0.285	0.341	2.353
387	0.407	1.336	0.330
388	0.972	1.433	3.416
389	0.318	1.088	1.227
390	0.577	1.849	1.743
391	0.470	1.632	3.115
392	0.945	1.115	1.054
393	0.498	0.382	0.907
394	0.406	1.545	0.227
395	0.407	0.514	0.239
396	0.769	1.440	1.751
397	0.613	1.390	1.347
398	0.418	0.319	3.001

Structure no.	Length (µm) of Ald4p-GFP structures		
	Reference	Reference with *FLO9* from SWORD	SWORD
399	0.338	0.550	3.189
400	0.402	0.237	2.189
401	0.775	1.342	0.664
402	0.534	1.197	0.372
403	0.587	2.154	0.491
404	1.443	1.090	0.315
405	0.835	0.194	0.298
406	0.932	0.585	0.904
407	0.861	1.114	1.569
408	0.328	0.606	0.763
409	1.038	0.303	2.619
410	0.140	1.702	3.102
411	0.406	0.550	0.625
412	0.425	1.103	0.220
413	1.118	0.762	2.181
414	0.830	1.483	3.765
415	0.245	0.824	0.564
416	1.336	1.060	0.225
417	1.534	0.477	2.776
418	1.223	0.756	4.105
419	0.332	0.767	2.675
420	0.220	1.873	2.863
421	0.377	1.216	1.912
422	0.273	0.430	2.548
423	0.215	0.637	1.823
424	0.480	0.775	1.770
425	0.484	0.482	0.590
426	0.355	1.692	0.629
427	0.630	0.287	1.381
428	0.235	1.137	0.413
429	0.318	0.782	0.143
430	0.299	0.230	1.337
431	0.390	0.154	2.266
432	0.408	0.180	0.428
433	1.036	0.161	0.462
434	2.205	0.438	0.183
435	0.900	1.506	4.029
436	0.261	0.599	1.933
437	1.254	0.896	2.938
438	0.353	0.431	2.363

Biology Open • Supplementary information

Structure no.	Length (μm) of Ald4p-GFP structures		
	Reference	Reference with *FLO9* from SWORD	SWORD
439	0.317	1.056	2.625
440	1.200	0.839	1.994
441	0.690	1.090	3.166
442	0.578	1.278	2.726
443	0.558	0.912	0.323
444	0.489	1.161	2.581
445	0.556	0.248	0.297
446	1.457	0.725	1.246
447	0.215	0.487	2.638
448	0.466	0.281	2.333
449	0.754	0.636	2.351
450	1.258	0.261	2.846
451	1.051	0.329	3.849
452	0.845	1.918	1.231
453	0.515	1.725	3.462
454	0.242	1.703	2.987
455	0.423	0.825	0.143
456	0.461	1.061	1.787
457	0.658	2.023	2.032
458	0.544	1.196	0.475
459	0.537	1.336	0.297
460	0.161	1.249	2.363
461	0.579	1.784	2.933
462	0.366	0.366	0.719
463	0.515	0.668	0.998
464	1.038	1.404	3.173
465	1.058	0.519	3.123
466	0.208	0.836	3.295
467	0.315	0.834	3.667
468	0.371	0.665	0.519
469	1.269	1.246	3.119
470	0.837	0.787	2.741
471	0.403	1.027	3.398
472	0.784	0.337	2.793
473	1.113	0.352	2.384
474	1.092	0.369	2.990
475	0.327	0.436	1.006
476	0.476	0.567	1.040
477	0.327	0.411	3.085
478	0.506	0.967	2.676

Structure no.	Length (μm) of Ald4p-GFP structures		
	Reference	Reference with *FLO9* from SWORD	SWORD
479	0.962	2.041	0.349
480	0.418	1.281	2.931
481	0.316	1.134	0.317
482	0.371	1.142	2.572
483	0.764	0.869	2.427
484	0.360	0.332	0.882
485	1.319	0.519	2.665
486	0.210	0.794	0.445
487	0.494	1.026	1.427
488	0.912	0.731	3.535
489	1.107	1.416	1.497
490	1.458	1.902	1.516
491	0.243	0.723	1.568
492	0.417	0.929	1.340
493	1.915	0.720	1.451
494	0.469	0.337	3.186
495	1.389	0.513	1.620
496	0.288	1.334	1.397
497	0.556	0.140	1.424
498	0.390	0.616	2.649
499	0.599	0.484	0.134
500	0.785	0.690	1.333
501	0.202	0.215	1.333
502	0.581	0.572	1.098
503	0.231	1.346	2.206
504	0.516	1.278	1.161
505	0.395	1.204	0.151
506	0.678	0.328	2.078
507	0.161	1.590	0.143
508	0.916	1.001	1.744
509	0.465	0.199	0.619
510	0.593	0.821	0.180
511	1.039	0.736	0.202
512	0.290	1.243	0.265
513	0.476	0.643	0.794
514	0.190	0.957	1.316
515	1.117	0.875	0.275
516	1.005	0.563	1.138
517	0.316	1.112	1.462
518	1.892	0.623	0.335

Structure no.	Length (μm) of Ald4p-GFP structures		
	Reference	Reference with *FLO9* from SWORD	SWORD
519	1.313	0.398	0.646
520		2.139	3.710
521		0.520	0.458
522		1.094	3.817
523		0.478	1.810
524		0.140	1.138
525		1.325	2.376
526		0.476	1.289
527		0.259	0.654
528		0.789	0.627
529		0.668	0.143
530		0.749	0.154
531		1.087	2.719
532		0.648	3.881
533		1.354	2.474
534		0.357	0.918
535		1.093	1.062
536		0.883	0.561
537		0.745	0.441
538		1.914	0.760
539		0.812	0.577
540		0.542	0.771
541		0.656	3.699
542		0.645	5.085
543		1.687	2.744
544		0.457	3.324
545		0.510	3.620
546		0.871	1.248
547		0.830	1.115
548		0.916	1.087
549		1.539	0.576
550		1.185	4.967
551		0.823	3.700
552		0.706	1.439
553		0.166	3.460
554		0.385	0.201
555		1.275	3.440
556		0.281	2.033
557		1.306	1.436
558		0.255	1.893

Biology Open • Supplementary information

Structure no.	Length (µm) of Ald4p-GFP structures		
	Reference	Reference with *FLO9* from SWORD	SWORD
559		1.501	3.297
560		1.596	1.029
561		0.212	1.007
562		1.273	2.696
563		0.966	1.478
564		0.695	2.062
565		0.565	2.202
566		0.499	3.296
567		0.301	3.129
568		0.227	1.420
569		1.073	3.649
570		1.935	3.001
571		0.199	0.987
572		0.382	1.242
573		0.231	3.766
574		0.993	1.686
575		0.763	0.899
576		2.087	3.541
577		0.561	3.061
578		0.427	3.205
579		0.198	2.981
580		0.394	1.629
581		0.855	1.157
582		2.319	1.519
583		0.649	2.186
584		0.871	3.584
585		1.066	3.116
586		0.644	0.623
587		0.401	1.109
588		0.586	0.801
589		0.383	2.732
590		1.493	0.256
591		1.192	0.853
592		1.445	1.497
593		0.898	1.560
594		2.403	0.933
595		0.547	0.447
596		0.675	2.939
597		0.780	1.496
598		0.790	0.490

Biology Open • Supplementary information

Structure no.	Length (μm) of Ald4p-GFP structures		
	Reference	Reference with *FLO9* from SWORD	SWORD
599		1.089	1.252
600		1.502	4.014
601		1.203	2.197
602		1.392	1.047
603		0.611	3.511
604		0.449	1.513
605		0.234	2.760
606		0.796	2.427
607		1.911	3.580
608		0.811	1.271
609		1.205	5.324
610		0.990	3.384
611		1.772	1.970
612		0.862	0.433
613		1.495	3.221
614		0.550	0.627
615		0.193	1.600
616		0.154	0.253
617		2.166	2.245
618		0.925	2.142
619		0.456	
620		0.588	
621		1.362	
622		0.631	
623		1.768	
624		1.467	
625		1.042	
626		0.393	
627		0.881	
628		0.736	
629		1.822	
630		1.140	
631		0.938	
632		0.570	
633		1.248	
634		0.410	
635		1.502	
636		0.296	
637		0.706	
638		0.891	

Structure no.	Length (μm) of Ald4p-GFP structures		
	Reference	Reference with *FLO9* from SWORD	SWORD
639		1.222	
640		0.587	
641		0.383	
642		1.188	
643		0.184	
644		1.279	
645		0.882	
646		0.409	
647		0.706	
648		1.364	
649		1.487	
650		0.964	
651		1.107	
652		0.588	
653		0.143	
654		0.352	
655		0.531	
656		0.777	
657		1.536	
658		0.842	
659		0.482	
660		0.590	
661		0.842	
662		0.703	
663		0.492	
664		1.011	
665		2.377	
666		1.956	
667		0.283	
668		1.616	
669		1.320	
670		0.180	
671		0.606	
672		0.514	
673		0.704	
674		0.366	
675		1.348	
676		0.413	
677		1.026	
678		0.752	

Structure no.	Length (µm) of Ald4p-GFP structures		
	Reference	Reference with *FLO9* from SWORD	SWORD
679		0.703	
680		0.426	
681		1.110	
682		0.337	
683		0.585	
684		1.002	
685		1.552	
686		1.591	
687		0.523	
688		0.327	
689		0.863	
690		0.798	
691		0.392	
692		0.618	
693		1.413	
694		0.333	
695		0.394	
696		1.095	
697		0.748	
698		0.453	
699		0.906	
700		0.968	
701		1.642	
702		0.317	
703		0.216	
704		0.151	
705		0.178	
706		0.203	
707		2.177	
708		1.493	
709		1.190	
710		1.088	
711		0.262	
712		0.270	
713		2.665	
714		0.437	
715		0.904	
716		1.700	
Total number of structures analyzed	**519**	**716**	**618**

Biology Open • Supplementary information
